# Multimodal diffusion for joint design of protein sequence and structure

**DOI:** 10.1002/pro.70340

**Published:** 2025-11-14

**Authors:** Shaowen Zhu, Siddhant Gulati, Yuxuan Liu, Siddhi Kotnis, Qing Sun, Yang Shen

**Affiliations:** ^1^ Department of Electrical and Computer Engineering Texas A&M University College Station Texas USA; ^2^ Department of Chemical Engineering Texas A&M University College Station Texas USA; ^3^ Interdisciplinary Graduate Program in Genetics and Genomics Texas A&M University College Station Texas USA

**Keywords:** diffusion models, generative models, machine learning, protein design

## Abstract

Computational design of functional proteins is of both fundamental and applied interest. This study introduces a generative framework for co‐designing protein sequence and structure in a unified process by modeling their joint distribution, with the goal of enabling cross‐modality interactions toward coherent and functional designs. Each residue is represented by three distinct modalities (type, position, and orientation) and modeled using dedicated diffusion processes: multinomial for types, Cartesian for positions, and special orthogonal group SO(3) for orientations. To couple these modalities, we propose a unified architecture, ReverseNet, which employs a shared graph attention encoder to integrate multimodal information and separate projectors to predict each modality. We benchmark our models, JointDiff and JointDiff‐x, on unconditional monomer design and conditional motif scaffolding tasks. Compared to two‐stage design models that generate sequence and structure separately, our models produce monomer structures with comparable or better designability, while currently lagging in sequence quality and motif scaffolding performance based on computational metrics. However, they are 1–2 orders of magnitude faster and support rapid iterative improvements through classifier‐guided sampling. To complement computational evaluations, we experimentally validate our approach through a case study on green fluorescent protein (GFP) design. Several novel, evolutionarily distant variants generated by our models exhibit measurable fluorescence, confirming functional activity. These results demonstrate the feasibility of joint sequence–structure generation and establish a foundation to accelerate functional protein design in future applications. Codes, data, and trained models are accessible at https://github.com/Shen-Lab/JointDiff.

## 1 INTRODUCTION

Proteins are vital for the functioning of living organisms, facilitating a wide variety of cellular processes through their various functions (Alberts, 2015). These functional macromolecules consist of one‐dimensional (1D) sequences of amino acids that intricately fold into distinct three‐dimensional (3D) structures, ultimately dictating their specific functions (Branden & Tooze, 2012). However, even with 10^8^–10^10^ naturally evolved proteins already sequenced (Richardson et al., 2023; The UniProt Consortium, 2019), the massive space of functional proteins remains largely unexplored (Huang et al., 2016), which presents opportunities for designing and engineering proteins with desired functions, such as catalysis (Röthlisberger et al., 2008), vaccination (Correia et al., 2014), immunotherapy (Silva et al., 2019), and biosensing (Ibraheem & Campbell, 2010).

Traditional experimental pipelines (Arnold, 1998; Dougherty & Arnold, 2009; Romero & Arnold, 2009) for protein design utilize directed evolution. This iterative process of mutagenesis and filtering, despite its success, demands costly and time‐consuming lab characterization, which bottlenecks throughput (Wittmann et al., 2021). Moreover, as the process starts with known wild types and continues with the best variants from previous rounds, the exploration of the protein space is often restricted to local neighborhoods of known proteins as opposed to de novo design, thereby limiting its potential (Huang et al., 2016).

Computational methods for protein design harness computing power to accelerate design throughput. These methods fall into two main categories based on their rationale: principle‐driven optimization and data‐driven machine learning.

Principle‐driven methods typically estimate energy scores from protein sequence and structure based on physical principles. Specifically they search for sequences and structures that optimize folding energy to stabilize a backbone structure (Dahiyat & Mayo, 1997; Kuhlman et al., 2003) or optimize binding energy to design a binder (Lippow et al., 2007). They can also start with a function motif and design the rest (Correia et al., 2010; Jiang et al., 2008; Silva et al., 2016; Yang et al., 2021). Meanwhile, data‐driven methods leverage machine learning, particularly deep generative models, along with growing datasets, to estimate sample distributions. Generative adversarial networks and (variational) encoder‐decoder models are first applied to learn protein sequence distributions conditioned on structural folds (Cao et al., 2021; Greener et al., 2018; Karimi et al., 2020), protein families (Madani et al., 2023; Shin et al., 2021), gene ontology terms (Cao, 2021), and textual function descriptions (Liu et al., 2023).

Diffusion models (Ho et al., 2020; Sohl‐Dickstein et al., 2015; Song et al., 2020) have been adopted to learn protein structure distributions, thanks to their strong performance in synthesizing images and point clouds. For instance, SMCDiff (Trippe et al., 2023), FoldingDiff (Wu et al., 2024), and LatentDiff (Fu et al., 2023) operate in the Cartesian, torsional, and latent spaces, respectively. Chroma (Ingraham et al., 2023) introduced a programmable design pipeline to steer structure generation under desired conditions. RFdiffusion (Watson et al., 2023) fine‐tunes the RoseTTAFold structure prediction model (Baek et al., 2021) for protein structure denoising tasks. Recent models such as MultiFlow (Campbell et al., 2024) based on flow matching have extended generative capabilities to jointly model protein sequence and structure, incorporating diffusion mechanisms for discrete amino acid types.

In parallel, large language models have been increasingly adopted for protein design. Early efforts focused on learning and generating protein sequences (Madani et al., 2023), which are inherently discrete in amino acid types. These models have since been extended to capture both sequence and structural information (Heinzinger et al., 2024; Su et al., 2024), facilitated by innovations such as FoldSeek (van Kempen et al., 2024), which introduces structural “alphabets” to represent continuous 3D structures as token sequences. Most recently, ESM3 (Hayes et al., 2025) has emerged as a multimodal generative language model capable of learning distributions over protein sequence, structure, and function simultaneously, demonstrating promising potential for programmable protein design.

Despite progress in designing protein sequences or structures using generative models, functional protein design requires sequence and structure co‐design. Designing sequences alone faces challenges in learning complex sequence–function relationships in a vast sequence space, which could benefit from the more conserved structure to regularize and explain models. Structure design alone suffers from limited resolution and functional sensitivity, which could be improved by incorporating sequence information.

Interestingly, many methods find protein sequence–structure co‐design (protein co‐design) empirically inferior to a two‐stage approach. They either design protein structures first and then design protein sequences for those structures (such as Chroma followed by a Potts model and RFdiffusion followed by ProteinMPNN [Dauparas et al., 2022] or LigandMPNN [Dauparas et al., 2025]) or vice versa (such as ProteinGenerator [Lisanza et al., 2024] using sequence denoising followed by RoseTTAFold structure updates). Even ESM3, a multimodal generative language model that learns joint distributions over protein sequence, structure, and function, does not generate multiple modalities simultaneously. Instead, it adopts a “chain‐of‐thought” approach across modalities. For example, when designing green fluorescent proteins (GFPs) conditioned on a functional motif, ESM3 first generates secondary structure tokens, followed by structure tokens, and finally the amino acid sequence. This sequential modality generation highlights ongoing challenges in achieving truly integrated co‐design.

In contrast to two‐stage prior work such as Chroma and ProteinGenerator, which alternate sequence and structure updates using separate networks and model one modality before conditioning on the other, in this study we propose a generative framework that directly learns and samples the joint distribution of sequence and structure. It employs a unified architecture ReverseNet to simultaneously update all modalities. This design enables integrated, multimodal diffusion within a single framework, which is not possible in methods that rely on alternating updates across separate networks. By learning their joint distribution, the framework establishes a foundation for richer cross‐modality interactions, with the goal of enabling coherent and functionally plausible protein designs.

**FIGURE 1:**
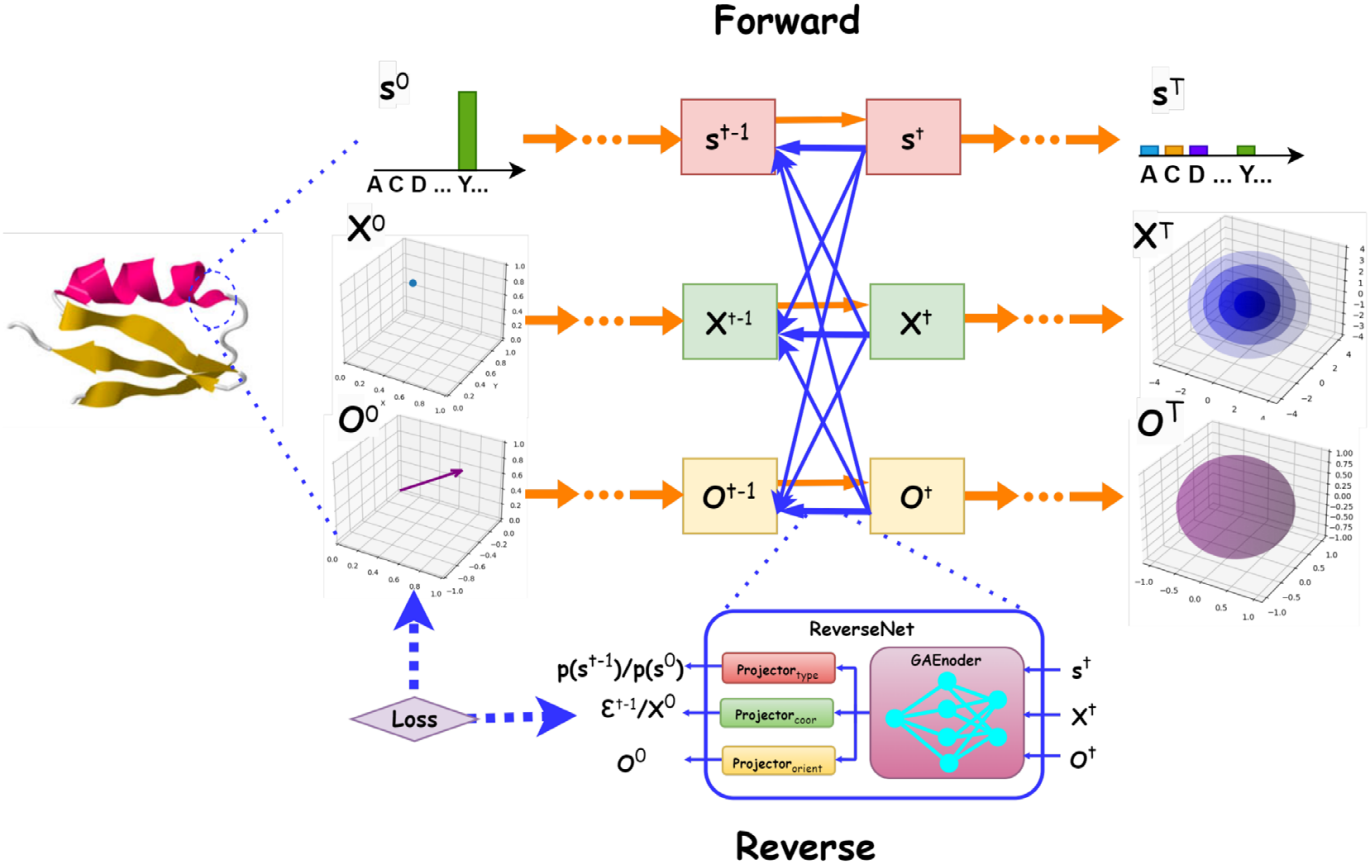
Illustration of the forward and reverse processes in JointDiff/JointDiff‐x. Protein sequence and backbone structure are described by three modalities at each residue: amino acid types, residue positions (C_α_ coordinates), and residue‐frame orientations. In the forward diffusion process, independent noise is added to each modality to progressively corrupt the data. In the reverse process, an SO(3)‐invariant graph attention encoder aggregates multimodal information to predict, resample sequences, and update structures in an SO(3)‐equivariant manner. It extracts rotation‐ and translation‐invariant node and edge features from all modalities and processes them through graph attention blocks with modality‐specific projectors (including an SE(3)‐equivariant coordinates projector), enabling unified denoising of all modalities.

We represent protein sequence and (backbone) structure as three modalities at each residue: discrete amino acid type, continuous rigid‐frame position in Cartesian space, and continuous rigid‐frame orientation in the special orthogonal group SO(3). To construct a multimodal diffusion framework, we integrate multinomial diffusion (Hoogeboom et al., 2021), Denoising Diffusion Probabilistic Models (DDPM) (Ho et al., 2020), and SO(3) diffusion (Leach et al., 2022) for the three modalities, respectively. As shown in Figure 1, we link the three diffusion processes in reverse processes through a ReverseNet architecture, which includes a common graph attention encoder (GAEncoder) with shared 3‐modality input and individualized 1‐modality projections. We use two types of losses to train our diffusion models, noise prediction (ε‐prediction) and ground‐truth prediction (*x*_0_‐prediction) that allows for additional structure regularization. These lead to two variants: JointDiff and JointDiff‐x.

Our models generate protein sequence–structure pairs simultaneously and are benchmarked extensively on both unconditional monomer design and conditional motif scaffolding. We also evaluate them across computational metrics such as sequence and structure self‐consistency, diversity, and novelty, as well as sequence–structure cross‐consistency. While our models generate highly designable monomer structures efficiently, they currently lag behind in sequence quality and motif scaffolding performance based on computational metrics. However, their speed enables rapid improvement in design quality through guided sampling, as demonstrated in case studies.

Building on the motif scaffolding capabilities of our model, we conduct a case study on the computational design and experimental validation of GFPs. Several evolutionarily distant variants exhibit measurable fluorescence, establishing a promising foundation for future rounds of optimization and functional protein design.

## 2 METHODS

In this section, we describe the design and implementation of our multimodal diffusion framework for joint protein sequence–structure generation. At the core of our approach is the ReverseNet architecture, which couples three modality‐specific diffusion processes (multinomial, Cartesian, and rotational/SO(3)) through a shared GAEncoder. We begin by formalizing the representation of protein sequence and structure, then introduce the JointDiff and JointDiff‐x models, detailing their architecture, diffusion mechanisms, training objectives, and structure‐aware losses. We further describe our strategies for motif scaffolding, dataset construction, and model training. Finally, we present auxiliary components including ConfidenceNet for ranking generated designs, classifier‐guided sampling for iterative, targeted generation, a computational pipeline for designing and selecting GFPs, and an experimental pipeline for validating their functional activity.

### 2.1 Protein description and notation

In this study we use italicized letters for scalar variables (lowercase) and bold roman letters for vectors (lowercase) and matrices (uppercase).

Protein sequence is often described as an amino acid character sequence $\left[aa_{1},\ldots ,aa_{L}\right]$ of length L, where $aa_{i}\in w$ refers to the amino acid at position *i* (i.e., residue *i*) and the alphabet w contains 20 letters for 20 standard amino acid types, that is, ∣w∣=20. We use one‐hot encoding for each residue $s_{i}=\text{OneHot}\left(aa_{i}\right)\in {\left\{0,1\right\}}^{L\times 20}$ and thus represent each sequence as $S=\left[s_{1},\ldots ,s_{L}\right]\in {\left\{0,1\right\}}^{L\times 20}$.

Protein structure is often described as an atom cloud of Cartesian coordinates under residue topologies. In this study we focus on protein backbone structure of four heavy atoms (N, C_α_, C, O) per residue. Instead of directly representing protein backbone heavy‐atom cloud as a 4*L* × 3 matrix, we follow AlphaFold2 to reduce the degree of freedom. On the premise that the distances and angles between the backbone heavy atoms in each residue are relatively fixed, each residue can be approximated by a rigid‐body residue frame and described by coordinates of C_α_ and orientation of the frame. In our work, we incorporated both sequence and structure, enabling accurate backbone recovery using residue frames together with ideal geometric constraints of different amino acids. Accordingly, we describe the backbone structure as $T=\mathrm{bb}\left(X_{C_{\alpha }},O,S\right)$, while $X_{C_{\alpha }}=\left[x_{1},\ldots ,x_{L}\right]\in {\mathbb{R}}^{L\times 3}$ are the C_α_ coordinates, $O=\left[O_{1},\ldots ,O_{L}\right]\in {\left(\mathrm{SO}\left(3\right)\right)}^{L}$ are the frame orientation vectors, and $\mathrm{bb}\left(\cdot \right)$ denotes the backbone reconstructed from frame positions, frame orientations and reference geometric constraints based on the sequence. The coarse‐grained backbone structure, together with the sequence information, can be “backmapped” to all‐atom structures by force‐field simulation (Brooks et al., 2009) or machine learning (Jumper et al., 2021; Yang & Gómez‐Bombarelli, 2023).

### 2.2 JointDiff: multimodal joint diffusion

Our goal is to learn the joint distribution of protein sequences and structures $p\left(S,T\right)$. Here sequence and structure are described as $S=\left[s_{1},\ldots ,s_{L}\right]\in {\left\{0,1\right\}}^{L\times 20}$ and $T=\mathrm{bb}\left(X_{C_{\alpha }},O,S\right)$ (backbone structure reconstructed from residue‐frame positions and orientations) as defined in Section 2.1. Three distinct *data modalities* are involved for each residue j: amino acid type $s_{j}$ in a discrete one‐hot vector, coordinates $x_{j}$ in continuous Cartesian space, and orientations $o_{j}$ in a special orthogonal group $\mathrm{SO}\left(3\right)$ (Figure 1).

As introduced in Section 1, our model (JointDiff) jointly learns the distribution over protein sequences and structures by integrating three diffusion mechanisms for joint diffusion: multinomial diffusion (Hoogeboom et al., 2021) for amino acid types, DDPM (Ho et al., 2020) for residue‐frame positions (C_α_ coordinates), and SO(3) diffusion (Leach et al., 2022) for residue‐frame orientations. This was inspired by DiffAb (Luo et al., 2022) for antigen‐specific antibody CDR design. As Figure 1 shows, these modalities employ distinct noises in the forward processes to corrupt native protein data and are coupled in the reverse processes via a shared score network (ReverseNet) architecture.

#### 2.2.1 Graph attention encoder across modalities

Before detailing each modality‐specific diffusion, we briefly describe the core component of our ReverseNet: the GAEncoder which jointly processes the three modalities in a geometrically consistent manner. At each time step *t*, we reconstruct the backbone structure from the current noisy inputs—sequence $S^{t}$, C_α_ positions $X_{C_{\alpha }}^{t}$, and orientations $O^{t}$—and represent the protein as a graph. This graph‐based representation ensures invariance to global rigid‐body transformations, which is critical for modeling protein geometry.

The GAEncoder extracts node‐level and edge‐level features that are invariant to rotations and translations. Node features include amino acid identity embeddings, local coordinate frames derived from backbone atoms, and dihedral angles. Edge features include pairwise amino acid identities, relative spatial positions, distances, and backbone geometry. These features are passed through a series of graph attention blocks (GABlocks), which aggregate contextual information across the protein graph.

After aggregation, modality‐specific multilayer perceptron (MLP) projectors update each modality separately. To maintain rotational equivariance across denoising steps, predicted positions and orientations are aligned with those from the previous step using reference frames. This design allows the model to simultaneously denoise and update all modalities in a unified framework.

Detailed pseudocode for GAEncoder, including NodeEmbed, PairEmbed, and GABlock, is provided in Section S2.1, Supporting Information S1.

We now describe the diffusion processes for each modality in detail.

#### 2.2.2 Multinomial diffusion for protein sequence

Protein sequence generation was built by adapting the multinomial diffusion (Hoogeboom et al., 2021) to amino acid types. In the forward process of $S^{t}$ (where t=0,…,T), sequence $S^{0}$ at t=0 is initialized as the ground‐truth sequence, and at each time step *t*, the sequence sampled from the previous step $S^{t-1}$ is injected with a noise of multinomial distribution for each position *j* by resampling amino acid over the 20 types. In the reverse process, the GAEncoder‐based ReverseNet would collect all data modalities at the current step and update the joint representation accordingly. An MLP‐based projector with the softmax layer is used to predict the multinomial distribution of the amino acid types for the next step. The detailed process is shown in Section S1.1, Supporting Information S1. The loss function for sequence diffusion is the Kullback‐Leibler divergence (KL‐divergence) between two distributions about $S^{t-1}$: the implicit posterior $q\left(S^{t-1}|S^{t},S^{0}\right)$ and the projector‐predicted distribution $p\left(S^{t-1}|S^{t},X_{C_{\alpha }}^{t},O^{t}\right)$, as Equation (1) shows.

(1) $$L_{\text{type}}^{t}=\frac{1}{L}\sum_{j=1}^{L}D_{\mathrm{KL}}\left(q\left(s_{j}^{t-1}|s_{j}^{t},s_{j}^{0}\right)\|p(s_{j}^{t-1}|S^{t},X_{C_{\alpha }}^{t},O^{t})\right).$$

#### 2.2.3 DDPM for frame positions

The generation of frame positions (C_α_ coordinates), treated as 3D point clouds, is modeled by DDPM (Ho et al., 2020). Prior to DDPM, centralizing and scaling original coordinates is found to impact its performances. The scaling weight (sw) is treated as a hyperparameter ranging from 0.01 to 1.0. A protein length‐dependent version is also tested: $1/\mathrm{sw}=c\times \left(a\times L+b\right)$, where *a* and *b* are from linear regression of standard deviations of C_α_ coordinates as a function of protein length *L* (Figure S1).

During DDPM, the forward process introduces Gaussian noise iteratively to the ground‐truth, native coordinates following a predefined scheduler. In the reverse process, a neural network $G\left(\cdot \right)$, including GAEncoder shared across all three modalities and an MLP projector for positions, takes current noisy data $\left(S^{t},X_{C_{\alpha }}^{t},O^{t}\right)$ and predicts $G\left(S^{t},X_{C_{\alpha }}^{t},O^{t}\right)$ for coordiante denoising. The DDPM process is detailed in Section S1.2, Supporting Information S1. The loss function for C_α_ coordinates diffusion is the Mean Squared Error (MSE) in noise prediction (also called ε‐prediction loss) as follows.

(2) $$\begin{array}{c} L_{\text{coor}}^{t}=\frac{1}{L}\sum_{j=1}^{L}∥\epsilon_{j}-G\left(S^{t},X_{C_{\alpha }}^{t},O^{t}\right)\left[j\right]{∥}^{2}, \end{array}$$

where ∥⋅∥ denotes L2 norm.

#### 2.2.4 SO(3) diffusion for frame orientations

The generation of frame orientations, represented with SO(3) elements, follows SO(3) diffusion (Leach et al., 2022). The forward process uses isotropic Gaussian noise to iteratively perturb the native orientations. In the reverse process, a neural network $H\left(\cdot \right)$ (again a shared GAEncoder across all three modalities and a dedicated projector to orientations) predicts the mean orientation at step t−1 given the *complete* state (including sequence **S** and structure **T**) at step *t*.

With the predicted orientation aligned with the ground‐truth orientation $O^{0}$, the loss function for training the orientation diffusion is defined as the Frobenius norm between ${\left(O_{j}^{0}\right)}^{T}{\hat{O}}_{j}^{t-1}$ and the identity matrix I:

(3) $$\begin{array}{c} L_{\text{orient}}^{t}=\frac{1}{L}\sum_{j=1}^{L}∥{\left(O_{j}^{0}\right)}^{T}{\hat{O}}_{j}^{t-1}-I{∥}_{F}^{2}, \end{array}$$

where ${\hat{O}}_{j}^{t-1}=H\left(S^{t},X_{C_{\alpha }}^{t},O^{t}\right)\left[j\right]$. More details about the SO(3) diffusion are included in Section S1.3, Supporting Information S1.

#### 2.2.5 Multimodal diffusion (default JointDiff )

As shown above, the three diffusions corresponding to the three protein data modalities are distinct yet interconnected: each noise prediction depends on the complete multimodal state at time *t*. Accordingly, their neural network estimators, featuring a shared GAEncoder and modality‐specific projectors, were jointly trained using the sum of the three individual losses, forming the overall multimodal diffusion loss:

(4) $$\begin{array}{c} L_{\text{multi}}=E_{t∼\text{Uniform}\left(1,\ldots ,T\right)}\left[L_{\text{type}}^{t}+L_{\text{coor}}^{t}+L_{\text{orient}}^{t}\right], \end{array}$$

where *t* is randomly sampled from integers in $\left[1,\cdots ,T\right]$.

### 2.3 JointDiff‐x: ground‐truth prediction and additional structure losses

To further improve the alignment of the three diffusion processes, we tested changing noise prediction (ε‐prediction) to ground‐truth prediction (*x*_0_‐prediction), which allows for additional losses to enforce desired geometry on the predicted structures. The resulting models are thus dubbed JointDiff‐x.

#### 2.3.1 x_0_‐prediction losses

To enable the neural network estimators in the reverse process to directly predict the unperturbed sequence $S^{0}$ and structure (frames) $\left(X_{C_{\alpha }}^{0},O^{0}\right)$ at time 0 based on the perturbed state at time *t*, we modified the JointDiff multimodal loss function (Equation 4) as follows: we retained the orientation loss in Equation (3) and replaced the losses for amino acid type and frame position (i.e., C_α_ coordinates) in Equations (1) and (2) with Equations (5) and (6), respectively:

(5) $$\begin{array}{c} L_{\text{type}}^{t}=\frac{1}{L}\sum_{j=1}^{L}\text{CrossEntropy}\left(p\left(s_{j}^{0}\;|\;S^{t},X_{C_{\alpha }}^{t},O^{t}\right),s_{j}^{0}\right). \end{array}$$

(6) $$\begin{array}{c} L_{\text{coor}}^{t}=\frac{1}{L}\sum_{j=1}^{L}∥x_{j}^{0}-G\left(S^{t},X_{C_{\alpha }}^{t},O^{t}\right)\left[j\right]{∥}^{2}. \end{array}$$

Above we used MSE as the default coordinate loss in JointDiff‐x. Additionally, we evaluated the SE(3)‐invariant Frame Aligned Point Error (FAPE) loss, originally introduced in AlphaFold2 (Jumper et al., 2021) (see Section S2.2, Supporting Information S1 for details).

During inference, that is, the reverse process, at each step *t* the estimated $S^{0}$, $X_{C_{\alpha }}^{0}$ and $O^{0}$ would be based on $S^{t}$, $X_{C_{\alpha }}^{t}$ and $O^{t}$ and correupted with noise to obtain $S^{t-1}$ and $X_{C_{\alpha }}^{t-1}$ and $O^{t-1}$ for the next step t−1. When t=1, the predictions are the final output. More details can be found in Section S2.3, Supporting Information S1.

#### 2.3.2 Structure regularization

With *x*_0_‐prediction models, we are directly predicting the ground‐truth samples instead of noises, making it possible to incorporate further regularization on such structure predictions. Inspired by AlphaFold2 (Jumper et al., 2021) and AlphaFold‐Multimer (Evans et al., 2021), we added structure regularizations in the loss function based on the following residue–residue pairwise distances.

Distance loss is to compare such pairwise distances in real values between the predicted C_α_ coordinates ${\hat{x}}^{0}$ and the native C_α_ coordinates $x^{0}$, using squared error (d‐sq) or absolute error (d‐abs):

(7) $$\begin{array}{l} L_{d-\mathrm{sq}}=\begin{array}{l} \frac{2}{L\left(L-1\right)}\sum_{i=1}^{L}\sum_{j=i+1}^{L}\max((\left\|{\hat{x}}_{i}^{0}-{\hat{x}}_{j}^{0}\right\|{-\;\left\|x_{i}^{0}-x_{j}^{0}\right\|)}^{2},\delta^{2}), \end{array}\\ L_{d-\mathrm{abs}}=\begin{array}{l} \frac{2}{L\left(L-1\right)}\sum_{i=1}^{L}\sum_{j=i+1}^{L}\max(\left|\left\|{\hat{x}}_{i}^{0}-{\hat{x}}_{j}^{0}\left\|-\right\|x_{i}^{0}-x_{j}^{0}\right\|\right|,\delta ). \end{array} \end{array}$$

Following AlphaFold2, the error in pairwise distances is clamped at *δ* to prevent the loss from being dominated by few outlier pairs, where *δ* is set at 20 Å by default and tested at 10 and 30 Å as well.

Distogram loss compares such pairwise distances as distributions over discretized bins (distograms), using a cross‐entropy loss as in AlphaFold2 (Jumper et al., 2021). Its goal is to encourage the learned representations to capture geometric information and assist structure prediction.

Clash loss penalizes C_α_ atom pairs that are closer than 3.6 Å (Li & Zhang, 2009), discouraging steric clashes and promoting physically plausible structures:

(8) $$\begin{array}{c} L_{\text{clash}}=\frac{1}{N_{\text{clash}}}\sum_{i=1}^{L}\sum_{j=i+1}^{L}\max\left(3.6-∥{\hat{x}}_{i}^{0}-{\hat{x}}_{j}^{0}∥,0\right), \end{array}$$

where $N_{\text{clash}}$ is the count of clashes and the loss would be zero when $N_{\text{clash}}=0$.

#### 2.3.3 Random masking and motif scaffolding

Building on the concept of masked prediction, which has proven effective in natural language processing (Devlin et al., 2019) and computer vision (He et al., 2022), we extended our training strategy to incorporate random masking for two complementary purposes: enhancing unconditional monomer design by modeling dependencies across protein regions, and enabling conditional motif scaffolding, where the goal is to design scaffolds that support predefined functional motifs (Wang et al., 2022). In this conditional setting, protein regions are divided into unmasked (motif) and masked (scaffold) segments, with the unmasked motifs serving as fixed context. During training, GAEncoder receives unperturbed ground‐truth motifs along with perturbed scaffold regions at time *t*, rather than the entire perturbed protein. Each of the three diffusion losses is applied to both motif and scaffold portions, with a designated “motif factor” scaling the contribution of the motif regions.

To implement this framework, we also trained JointDiff and JointDiff‐x using a random masking protocol following RFdiffusion. Each training sample had an 80% probability of undergoing masking, with the masking ratio uniformly sampled from $\left[0.2,1.0\right]$. This setup trained the models to generate structurally coherent scaffolds conditioned on the presence and position of functional motifs, allowing us to assess their capacity for context‐aware protein design in realistic scenarios. For monomer design, models were retrained from scratch with a motif factor of 0.

### 2.4 Dataset

We collected training and validation sets from CATH v. 4.2 (Sillitoe et al., 2019) following Fold2Seq (Cao et al., 2021). Due to computational resources, we retained single‐chain contiguous proteins whose lengths are between 20 and 200 and only contain 20 standard amino acids. The whole dataset was first split at the fold level into subsets (*a*), (*b*), and (*c*) with a fold ratio of 95%, 2.5% and 2.5%, respectively. For subset (*a*), we further split it into (*a*1), (*a*2), and (*a*3) at the protein‐sample level with a sample ratio of 95%, 2.5% and 2.5%, respectively. Finally we used (*a*1) as the training set and (*a*2) + (*b*) as the validation set where (*a*2) contains training folds and (*b*) consists of new folds. The training set contained 45,995 protein samples of 971 folds, whereas the validation set contained 4159 samples of 185 folds. The test set (*a*3) of new folds was not used as monomer design is unconditional.

We also obtained another training dataset from the AlphaFold Protein Structure Database (AFDB) (Varadi et al., 2024). Specifically, we downloaded the predictions of single‐chain proteomes for 48 organisms, comprising 564,446 samples, and filtered for sequences of up to 300 amino acids, yielding a final set of 245,712 samples. To design GFPs, we additionally collected a fine‐tuning dataset from InterPro (Hunter et al., 2009) focused on the GFP family (InterPro ID: IPR009017). We retained samples containing between 178 and 277 amino acids, corresponding to ±50 residues around our target protein size (227).

Besides monomer design, our models trained with random masking were also tested in the task of motif scaffolding. Twenty‐one single‐chain cases among 24 motif scaffolding benchmarks from the RFdiffusion study were tested.

### 2.5 Model training and inference

JointDiff models were trained in batches of 16 samples for up to a million iterations on four Tesla T4 graphics processing units (GPUs). In each iteration we uniformly sampled a *t* from [1, 100] for each sample and calculated the loss following Equation (4) (with additional losses of structure regularization for JointDiff‐x). Our models were trained with an Adam optimizer with PyTorch default hyperparameters except for initial learning rate and weight decaying. Checkpoints of the minimum validation loss were chosen for early stopping, where the validation loss functions are the same as the training losses except for the weight decay term. Hyperparameters including initial learning rate ({1E−5, 5E−5. 1E−4, 5E−4, 1E−3}), weight decaying ({0.0, 0.01, 0.05}), and diffusion steps ($T\in \left\{10,50,100,500,1000\right\}$) were tuned based on the validation losses as well. In the end initial learning rate was chosen to be 1E−4, weight decaying to be 0.0, and diffusion steps (*T*) to be 100.

During inference, we generated 10 samples for each of the 181 protein sizes between 20 and 200, which resulted in 1810 samples in total.

Detailed algorithms for training and inference can be found in Sections S2.2 and S2.3, Supporting Information S1, respectively.

### 2.6 Model evaluation

For monomer design we evaluated the models using the average values of the following metrics, computed over 1810 samples spanning 181 sizes ranging from 20 to 200. These metrics reflect the model's ability to learn and generate across three data modalities: sequence, frame positions, and frame orientations. Foldability captures sequence plausibility; designability assesses backbone structure quality by evaluating both frame positions and orientations; and sequence–structure cross‐consistency measures the compatibility across all three modalities.

#### 2.6.1 Sequence or structure self‐consistency

These metrics assess how “protein‐like” a designed sequence or structure is. For each generated sequence **S**, we evaluate foldability by predicting its structure $T^{\prime }$ using ESMFold, inverse designing it to sequence $S^{\prime }$ using ProteinMPNN, and computing sequence identity between S and $S^{\prime }$. For each generated structure T, we evaluate designability by generating 10 sequences using ProteinMPNN, predicting their structures using ESMFold, and computing the average template modeling score (TM‐score) and Cα root mean square deviation (Cα RMSD, hereafter referred to simply as RMSD) between these structures and **T**.

(9) $$\begin{array}{l} \text{Self–Consistenc}y_{\mathrm{seq}}\left(S\right)\\ =\mathrm{SI}\left(S,\text{MPNN}\left(\text{ESMFold}\left(S\right)\right)\right)\;\text{(Foldability).}\\ \text{Self–Consistenc}y_{\mathrm{str}}\left(T\right)\\ =\begin{array}{l} \mathrm{TM}–\text{score}\left(T,\text{ESMFold}\left(\text{MPNN}\left(T\right)\right)\right)\;\text{(Designability).} \end{array} \end{array}$$

#### 2.6.2 Sequence–structure cross‐consistency

These metrics assess the alignment between each design pair $\left(S,T\right)$. For structure‐to‐sequence consistency, we inverse fold T into 10 sequences using ProteinMPNN and compute average sequence identity with S. For sequence‐to‐structure consistency, we fold S into $T^{\prime }$ using ESMFold and compute TM‐score (or RMSD) with T.

(10) $$\begin{array}{l} \text{Cross–Consistenc}y_{\mathrm{str}2\mathrm{seq}}\left(S,T\right)\\ \;=\mathrm{SI}\left(S,\text{MPNN}\left(T\right)\right)\;\left(\mathrm{str}\to \mathrm{seq}\right).\\ \text{Cross–Consistenc}y_{\mathrm{seq}2\mathrm{str}}\left(S,T\right)\\ \;=\mathrm{TM}–\text{score}\left(\text{ESMFold}\left(S\right),T\right)\;\left(\mathrm{seq}\to \mathrm{str}\right). \end{array}$$

These in silico metrics are widely used in recent state‐of‐the‐art models. The designability of a structure reflects whether an amino acid sequence can be identified with current methods to fold into the structure. It has been used to assess structure generation in SMCDiff (Trippe et al., 2023), FoldingDiff (Wu et al., 2024), FrameDiff (Yim et al., 2023), Multiflow (Campbell et al., 2024), RFdiffusion (Watson et al., 2023), and so on. Importantly, designability has been found to correlate with experimental success (Watson et al., 2023), making it a valuable criterion to guide experimental prioritization in tasks such as motif scaffolding (Wang et al., 2022), homo‐oligomer design (Wicky et al., 2022), and binder design (Bennett et al., 2023).

Another key metric is sequence‐to‐structure cross‐consistency for the generated pair of sequence and structure, which assesses whether a designed sequence folds into its designed structure. This metric has also demonstrated correlation with experimental outcomes, as shown in Chroma (refolding analysis) (Ingraham et al., 2023) and ProteinGenerator (Lisanza et al., 2024).

Given that protein sequences and structures do not follow a strict one‐to‐one mapping and that structures are generally more conserved than sequences, we interpret these metrics using biologically informed and experimentally validated thresholds. For structure‐to‐sequence cross‐consistency, a sequence identity above 30% is considered adequate, as protein sequences above this threshold in the same family often share similar structures. The same 30% threshold in sequence similarity applies to sequence foldability. For structure self‐consistency (designability), an RMSD below 2 Å is often associated with experimental success (Watson et al., 2023) yet a TM‐score above 0.5, a less stringent criterion, is also used in some studies (Ingraham et al., 2023; Wu et al., 2024). The same thresholds in structure similarity apply to sequence‐to‐structure cross‐consistency.

#### 2.6.3 Speed

For fair comparison, all model inference jobs were run on a single Tesla T4 GPU (16 GB) with a batch size of 1 and time recorded.

#### 2.6.4 Sanity check

For sequence: percentage of residues that are part of repeats with length 5 or more (% 5‐repeats). For structure: number of atomic clashes (clash count). For sequence: percentage of residues that are part of repeats with length 5 or over (% 5‐repeats). For structure: number of clashing C_α_ atom pairs (clash count).

To save computational burden, 500 samples were randomly selected from all 1,810 generated samples to evaluate the following metrics.

#### 2.6.5 Diversity and novelty

Similar to FoldingDiff (Wu et al., 2024) and Multiflow (Campbell et al., 2024) we used the cluster portion and the maximum similarity to the training set as metrics for diversity and novelty, respectively. For diversity, we used MMSeqs2 (Steinegger & Söding, 2017) with an identity threshold of 0.7 to cluster generated sequences and FoldSeek (van Kempen et al., 2024) with a TM‐score threshold of 0.7 to cluster generated structures. Then we calculated the ratio of cluster count and sample count as a measure of diversity. For novelty, we clustered the union of training proteins and designed samples and set the sequence identity threshold at 0.3 and TM‐score threshold at 0.5. We calculated the portion of designs that did not share clusters with any training sample as a measure of novelty. Other thresholds were also applied.

For motif scaffolding, we used the same assessment metrics as above and focused on designability. Following RFdiffusion, success is defined by designability in RMSD below 2 Å and a success rate is adopted as the major metric. Another success criterion based on designability in TM score above 0.5 was also tested, which was less stringent.

### 2.7 Confidence model for ranking generated designs

Given the high diversity of samples produced by diffusion models, it is important to rank and filter generated designs effectively. A straightforward approach is to evaluate each sample using external metrics such as foldability, designability, and cross‐consistency. However, this process is computationally expensive, requiring GPU hours to run oracle models like ESMFold and ProteinMPNN.

To address this, we developed a ConfidenceNet to predict the quality of generated samples directly. The architecture of ConfidenceNet mirrors that of ReverseNet, the shared GAEncoder and the separate projection layers. Unlike ReverseNet, ConfidenceNet's projections predict design quality evaluated in the aforementioned metrics—specifically, sequence‐ and structure‐ self‐consistency (foldability and designability) and sequence–structure cross‐consistency.

For each sample, residue‐wise representations from the GAEncoder are aggregated using average pooling, followed by linear projection to predict quality scores. We implemented two variants of the confidence model: one trained as a regression task to predict continuous metric values, and another trained as a multi‐label binary classification task, where continuous values are thresholded into binary labels. For sequence‐centered metrics, a positive label corresponds to sequence identity above 0.3; for structure‐centered metrics, a positive label corresponds to C_α_ RMSD below 2.0 Å.

To train the confidence model, we collected metric values for all natural protein samples and additionally generated 50,000 synthetic samples with corresponding evaluations. Synthetic samples were clustered by sequence identity (thresholded at 0.3), and dataset splits were performed at the cluster level to ensure diversity. The combined dataset of natural and synthetic samples was used for training.

Since the scales of different metrics vary—for example, sequence identity ranges from 0 to 1, while RMSD can exceed 1—we also experimented with normalizing all metric values to the range $\left[-1,1\right]$ prior to training.

ConfidenceNet was evaluated in Table S1 and only used in the GFP design portion of the study.

### 2.8 Guided sampling toward desired properties

To bias generation toward specific properties (illustrated here by structural architectures in the CATH hierarchy), we implemented a guided sampling procedure for JointDiff‐x. Guidance was provided by a pretrained CATH classifier from Chroma (Ingraham et al., 2023), which operates on full backbone atoms. We reconstructed these atoms from JointDiff‐x outputs $\left({\hat{X}}_{C_{\alpha }}^{t},{\hat{O}}^{t}\right)$ in the reverse process at time *t*, evaluated the classifier, and backpropagated the negative log‐likelihood loss to the C_α_ coordinates. The resulting gradient was rescaled and applied to the clean prediction using a quadratic decay scheduler. Full algorithmic details are provided in Section S2.4, Supporting Information S1.

To benchmark efficiency, we compared JointDiff‐x and RFdiffusion under identical guidance conditions. Oxygen atoms required for RFdiffusion were placed deterministically prior to classifier evaluation. Guided RFdiffusion incorporated the classifier loss via its predefined interface for auxiliary potentials, using the same guidance weight and decay schedule.

To choose representative case studies, we selected 1000 domains from the CATH 4.2 database, estimated classifier accuracy across architecture categories, and chose four with high accuracy: CATH 1.10 (orthogonal bundle, mainly alpha, accuracy = 0.7564), CATH 2.60 (sandwich, mainly beta, accuracy = 0.7974), CATH 3.30 (two‐layer sandwich, alpha and beta, accuracy = 0.6887), and CATH 3.40 (three‐layer [aba] sandwich, alpha and beta, accuracy = 0.8757). For each architecture, we generated 40 structures of length 120 using both models, with and without classifier guidance. We recorded wall‐clock time per sample and tracked classifier‐assigned probabilities of the target architecture throughout generation.

### 2.9 Computational design and selection for green fluorescent proteins

To further evaluate functional design capabilities, we conducted a case study using GFPs. To promote diversity while preserving function, we selected three distinct GFP structural templates: avGFP R96A variant (protein data bank [PDB] ID:1QY3), superfolder GFP or sfGFP (PDB:2B3P), and extra‐superfolder GFP or extra‐superfolder green fluorescent proteins (PDB:5B61). We pretrained JointDiff‐x (FAPE variant, random masking, and motif factor MF = 1) on the AFDB dataset with up to 300 amino acids, fine‐tuned it on a curated GFP dataset (see the supplemental in silico assessment in Table S4), defined chromophore‐forming and functionally critical residues (positions 96, 58–71, and 222) as the motif for each template, and used JointDiff‐x to sample sequence–structure pairs that scaffold the motif. Designs were evaluated and selected using previously established in silico metrics (e.g., designability) along with additional filtering criteria, before selected designs were experimentally tested.

During inference, the motif was centralized at the origin, and scaffold regions were initialized from random noise. At each reverse diffusion step, the native motif along with the current scaffold were the input, while the entire protein (sequence, C_α_ positions, and frame orientations) was the output. To ensure SO(3) equivariance, the generated motif was aligned to the native motif, with the generated scaffold rigidly moved accordingly.

To guide sequence sampling toward evolutionarily plausible variants, we incorporated multiple sequence alignment (MSA)‐based guidance. Specifically, we derived a position‐specific scoring matrix (PSSM) from an MSA of wild‐type GFP generated using MMseqs2. The top 200 homologs were used to compute amino acid frequencies, which were log‐transformed to construct the PSSM. During sampling, model output logits were adjusted by adding a scaled PSSM term at each residue position. Seven PSSM bias weights were tested: 0.5,1,2,3,5,10,50, with 1000 sequence–structure pairs generated per setting. Among three templates and seven PSSM weights, 21,000 designs were generated.

To select among the 21,000 designs for experimental validation, we applied a multi‐stage filtering pipeline for structural plausibility, functional relevance, and biophysical stability:

- *Designability and foldability*: An in‐house confidence model as described in Section 2.7 was trained to quickly predict these previously defined assessment metrics without time‐consuming ESMFold, AlphaFold2, or ProteinMPNN. Only high‐confidence samples were retained.
- *Functional motif fidelity*: RMSD in central alpha helix and chromophore site (as introduced in ESM3) were computed for AlphaFold2‐predicted structures from designed sequences, against GFP templates to ensure preservation of key secondary structures and chromophore alignment.
- *Stability prediction*: Stability was predicted using the in silico evaluation of unfolding free energy with with unfolded states modeling (IEFFEUM) model (Lee & Park, 2025), and only sequences exceeding −1.5 (stability prediction result for avGFP) were kept.
- *Functional classification*: The Chroma CATH classifier was used to confirm correct structural architecture. Only designs classified into the GFP superfamily were retained.

After filtering, 32 sequences of various homology levels to their templates were prioritized as candidates for experimental validation next.

### 2.10 Experimental testing of fluorescence activities for selected designs

To validate the functional activity of our computationally designed GFP variants, we experimentally expressed them in *Escherichia coli* and quantified their fluorescence output under controlled conditions. The validation process included plasmid construction, transformation, protein expression, and fluorescence measurement, as detailed below.

#### 2.10.1 Plasmid construction

A bacterial expression vector—a modified pBbB8k plasmid backbone, with a strong BBa_J23119 constitutive promoter, BBa_B0034 ribosome binding site (RBS), BBa_B0015 terminator, pBBR1 origin of replication (ori), and kanamycin resistance gene (Kan^R^), was used to express all tested variants. The sequences for the promoter, RBS and terminator were obtained from the International Genetically Engineered Machine (iGEM) Registry of Standard Biological Parts. The GFP sequences were codon optimized for *E. coli* expression using Integrated DNA Technologies (IDT) Codon Optimization tool, and gene fragments for GFP variants were obtained from IDT (eBlocks Gene Fragments). The plasmid backbone and gene fragments were amplified using Phusion High‐Fidelity DNA Polymerase (Thermo Scientific) with compatible overhang sequences, cloned using Gibson assembly, and transformed into *E. coli* NEB 5‐alpha. The correctness of the sequence of the constructed plasmids was verified using Plasmidsaurus whole‐plasmid sequencing. *E. coli* NEB 5‐alpha was used for protein expression of the designed variants.

#### 2.10.2 Experimental validation of designs

**FIGURE 2:**
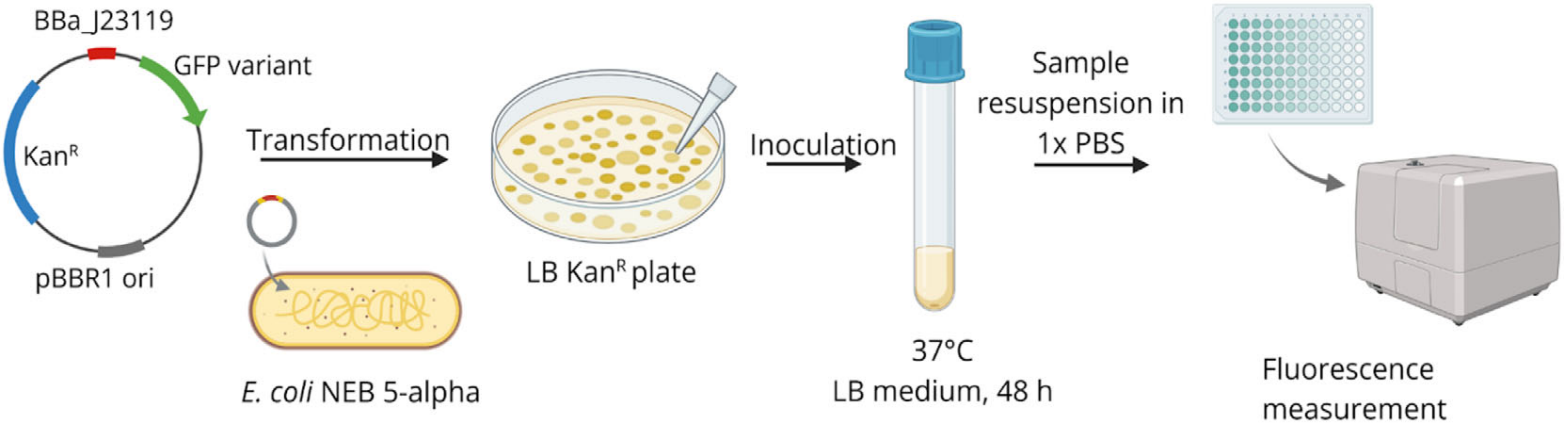
Schematic representation of the experimental method used to determine the fluorescence intensity of the designed green fluorescent protein (GFP) variants. LB, Lysogeny broth; PBS, phosphate buffered saline.

*E. coli* NEB 5‐alpha cells harboring plasmids containing the gene of interest were inoculated into a culture tube containing Lysogeny broth (LB)‐Miller medium (Fisher BioReagents) with 50 μg/mL kanamycin. The culture tubes were incubated at 37°C in a shaking incubator at 250 RPM. Samples were drawn at 24 and 48 h after inoculation. The obtained sample was centrifuged and the supernatant was discarded. The pellet was resuspended in 1× phosphate buffered saline (PBS), and transferred to a 96‐well plate (Black Side/Clear Bottom) for fluorescence and optical density (OD) measurements. The cell OD was measured at 600 nm. GFP fluorescence was measured using a BioTek Synergy H1 microplate reader (Agilent Technologies) under the following conditions: Excitation wavelength ($\lambda_{\mathrm{ex}}$)—485 nm, emission wavelength ($\lambda_{\mathrm{em}}$)—515 nm. A schematic representation of the experimental method is shown in Figure 2.

To analyze each sample, the OD_600_ and relative fluorescence intensity (RFU) values for 1× PBS were subtracted from each sample, and the obtained ratio of RFU and OD_600_ values was plotted. Wild‐type avGFP was used as a positive control, and *E. coli* cells harboring a plasmid without the GFP gene (empty vector [EV]) was used as a negative control. The fold‐change in fluorescence for each variant was reported after subtracting RFU/OD_600_ values for the EV from each sample.

## 3 RESULTS

In this section, we present a comprehensive evaluation of our multimodal diffusion frameworks, JointDiff and JointDiff‐x, across a range of protein design tasks. The results are organized to first examine the impact of architectural and training choices on monomer design quality, followed by comparisons with state‐of‐the‐art methods in terms of consistency, diversity, novelty, and speed. We then assess the efficiency of classifier‐guided sampling and the model's performance in motif scaffolding tasks. Finally, we demonstrate the practical potential of our framework through computational design and experimental validation of green fluorescent protein variants.

### 3.1 Impact of structure normalization on monomer design quality

To optimize structure representation for multimodal diffusion, we evaluated the impact of centering and scaling on JointDiff and JointDiff‐x. For JointDiff, centering improved designability by removing location bias, while scaling, especially length‐independent compression, yielded substantial gains in self‐consistency and cross‐consistency. A compression factor of sw=0.02 (50‐fold) proved most effective. Length‐dependent scaling offered no clear advantage. For JointDiff‐x, normalization had less impact due to its direct prediction of natural, unperturbed structures, but sw=0.02 was retained for consistency and performance. Detailed results and comparisons are provided in Table S2. The chosen JointDiff and JointDiff‐x models are also compared in details along with competing methods and natural proteins in the next subsection.

**FIGURE 3:**
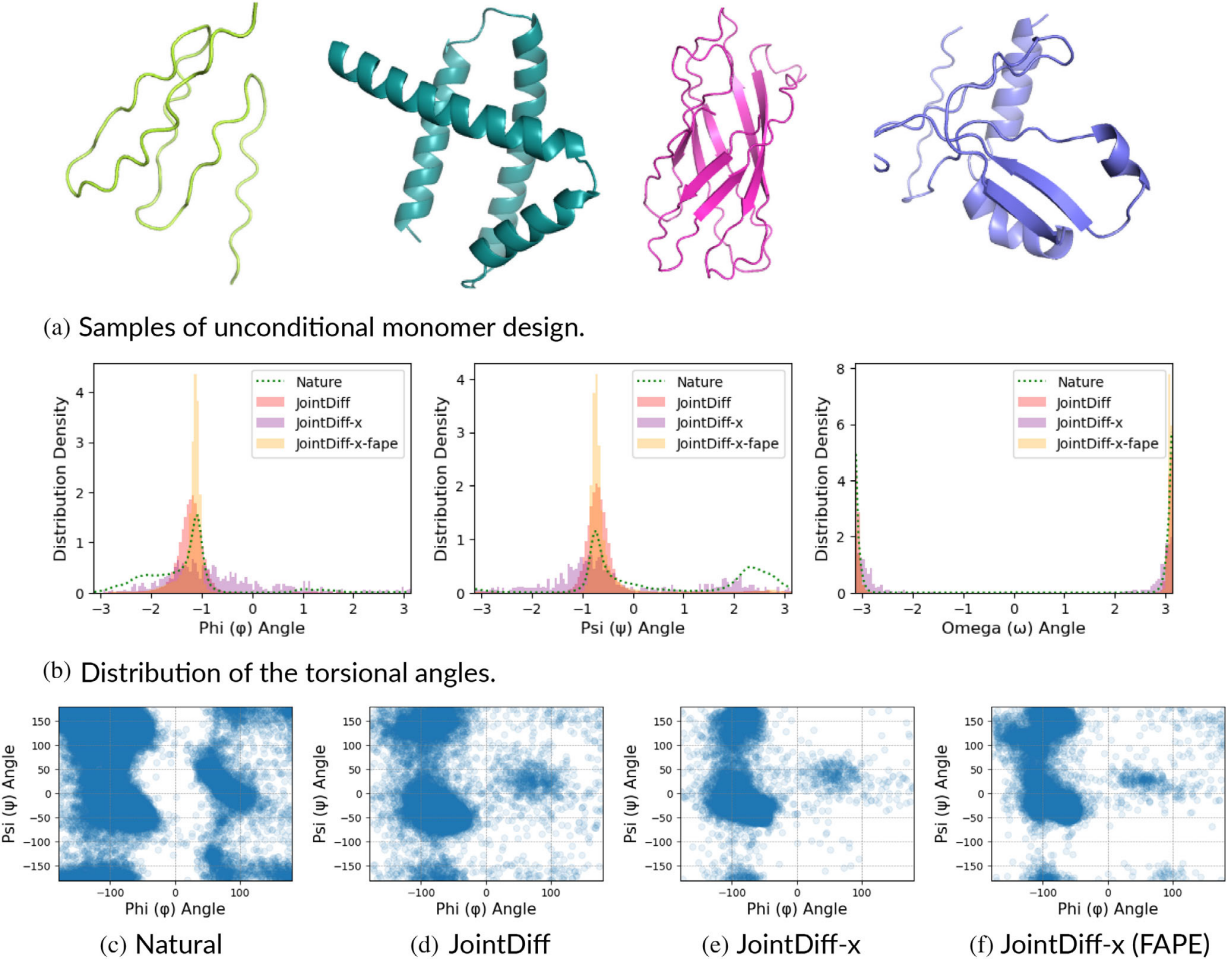
Visualizing 500 random backbones from training set and designs from JointDiff, JointDiff‐x, and JointDiff‐x (Frame Aligned Point Error [FAPE]) with coordinates centralized and scaled by sw=0.02 prior to position diffusion: (a) Four representatives of generated structures in distinct fold families; (b) Marginal distributions of three backbone torsional angles, natural or generated; and (c)–(f) Scatter plots of backbone (ϕ,ψ) pairs (Ramachandran plots) for natural and generated structures. (a) Samples of unconditional monomer design. (b) Distribution of the torsional angles. (c) Natural. (d) JointDiff. (e) JointDiff‐x. (f) JointDiff‐x (FAPE).

We also illustrate that JointDiff successfully generated structures resembling natural proteins, as representatives of various fold classes were visualized in Figure 3a, the torsional angle distributions of 500 random generated backbones versus 500 training natural backbones in Figure 3b, and Ramachandran plots for generated versus natural backbones in Figure 3d. ψ angles around $120^{{}^{\circ}}$ and left‐handed α‐helices were underrepresented in JointDiff‐generated backbones.

### 3.2 Impact of training losses on monomer design quality

We next explore the impact of training losses used for protein multimodal diffusion. In earlier JointDiff, we trained ReverseNet (GAEncoder shared across modalities and projectors individualized for each modality) to predict noise ε in the reverse process. This noise prediction (ε‐prediction) is equivalent to predict ground‐truth **x**_0_ at *t* = 0 but sometimes found empirically better than latter (Luo, 2022). Meanwhile, when unperturbed protein sequences and structures are predicted (*x*_0_‐prediction), they can be compared to ground‐truth values using different or additional losses than those in noise prediction. For instance, FAPE can replace MSE for the coordinate loss $L_{\text{coor}}$. Additional losses for predicting ground‐truth protein structures can be included, such as squared or absolute error in C_α_–C_α_ pairwise distances, penalties on clashes (Evans et al., 2021) and recovery of self‐supervised random masks. We refer to these models based off *x*_0_‐prediction as JointDiff‐x and summarize their performances in Table 1.

**TABLE 1:** Sequence or structure self‐consistency and sequence–structure cross‐consistency values for JointDiff‐x designs when models were trained with various losses: coordinate loss in the Mean Squared Error (MSE) or Frame Aligned Point Error (FAPE) form, pairwise distance (dist) loss with absolute (abs) or squared (sq) error, distogram (dg), clash, and random masking (rm). Whereas coordinate loss for ground‐truth prediction (*x*_0_‐prediction) can be either MSE or FAPE, additional structure losses can be included, such as those to constrain pairwise distances, distograms, and clashes. The random masking strategy can also be adopted. Boldfaced MSE and FAPE configurations were adopted for JointDiff‐x and JointDiff‐x (FAPE), respectively. Boldfaced entries highlight the top results for each evaluation metric.

JointDiff‐x configuration		Self‐consistency		Cross‐consistency		Clash
		seq (fold.)	str (design.)	str2seq	seq2str	str
		↑	TM↑/RMSD↓	↑	TM↑/RMSD↓	↓
coor. (MSE) loss	sw = 1.0	0.217	0.301/4.175	0.190	0.295/4.160	41.171
	sw = 0.1	0.225	0.597/2.784	0.154	0.280/4.281	123.254
	sw = 0.05	0.199	0.628/2.661	0.161	0.357/3.738	94.675
	sw = 0.02	0.205	0.640/2.603	0.163	0.366/3.717	93.895
	sw = 0.01	0.167	0.467/3.299	0.145	0.295/3.739	212.562
	length‐dep.	0.205	0.552/2.834	0.178	0.361/3.535	29.843
**coor. (MSE)**; ***sw* = 0.02**	dist(abs, *δ* = 20)	0.221	0.556/3.042	0.146	0.348/3.803	50.403
	dist(abs, *δ* = 10) + clash	0.234	0.611/2.706	0.188	0.356/3.783	7.330
	dist(abs, *δ* = 20) + clash	0.224	0.624/2.594	0.204	0.458/3.157	14.007
	dist(abs, *δ* = 30) + clash	0.197	0.645/2.586	0.142	0.338/3.798	5.249
	dist(sq, *δ* = 10) + clash	0.175	0.636/2.521	0.150	0.412/3.207	9.883
	dist(sq, *δ* = 20) + clash	0.224	0.630/2.652	0.175	0.326/3.727	13.209
	dist(sq, *δ* = 30) + clash	0.197	0.634/2.621	0.134	0.361/3.617	39.305
	**dist(abs, *δ* = 20) + dg + clash**	**0.255**	0.647/2.589	**0.205**	0.422/3.351	10.804
	dist(abs, *δ* = 20) + dg + clash + rm	0.234	0.518/3.007	0.197	**0.543**/**2.961**	8.131
**coor. (FAPE)**; ***sw* = 0.02**	dist(abs, *δ* = 20) + dg + clash	0.178	**0.784**/**1.792**	0.143	0.338/3.955	6.577
	**dist(abs**, *δ* = 20**) + dg + clash + rm**	0.208	0.737/2.157	0.166	0.357/3.790	10.504
	dist(abs, *δ* = 20) + dg + clash + rm + AFDB	0.200	0.722/2.228	0.160	0.346/3.790	**5.232**

Abbreviations: AFDB, AlphaFold Protein Structure Database; sw, scaling weight.

The first block in Table 1 shows that JointDiff‐x using ground‐truth prediction was less sensitive to normalization compared to JointDiff using noise prediction. The sw was chosen as sw=0.02. The ground‐truth prediction loss alone resulted in worse performances than noise prediction loss in JointDiff, especially in decreased sequence‐to‐structure cross‐consistency and increased structure clashes. However, the performance can be further regulated by introducing additional structure regularization losses as follows.

The second block in Table 1 evaluates the effects of incorporating additional structure losses such as distance, clash, and distogram as well as random masking in JointDiff‐x. The distance loss, based on absolute error in residue–residue pairwise geometry, reduced clash count substantially from 93.9 to 50.4, but also lowered structure designability (TM‐score) from 0.640 to 0.556. Notably, this TM‐score remained above the 0.5 threshold commonly used to define the same structural fold or to filter designs before experiments. Adding a clash loss further reduced clashes to 14.0 and restored designability to 0.624.

Adjusting the distance error cutoff *δ* revealed a trade‐off: a value of 20 Å offered a better balance than 10 or 30 Å, maintaining similar self‐consistency while improving sequence–structure cross‐consistency. This suggests an interplay between enforcing geometric accuracy and preserving sequence–structure alignment. While smaller cutoff may not enforce geometry enough to improve structure quality, larger cutoff considers structure outliers more and could reduce attention to sequence quality. Replacing the absolute error with squared error in the distance loss did not yield clear improvements.

Introducing a distogram loss, which enforces pairwise distances in a discretized format, further enhanced structure designability (TM‐score from 0.624 to 0.647 and RMSD from 2.594 to 2.589) and sequence foldability (from 0.224 to 0.255), though it slightly reduced sequence‐to‐structure cross‐consistency (TM‐score from 0.458 to 0.422 and RMSD from 3.157 to 3.351 Å).

Incorporating random masking—designed to capture dependencies across protein regions—yielded the best performance on sequence–to‐structure cross‐consistency (TM‐score of 0.543 and RMSD of 2.961 Å). However, it impaired the performance on other metrics, especially when designability in TM‐score dropped to 0.518 and that in RMSD increased to 3.007 Å. For a balanced view, we chose the configuration without random masking for JointDiff‐x when the coordinate loss is in the default MSE form. The resulting structure designability (TM‐score of 0.647) exceeded the 0.5 threshold used by Chroma and FoldingDiff, while sequence foldability reached 0.255, which was the highest among all tested configurations.

Finally, SE(3)‐invariant FAPE was tested as the position loss $L_{\text{coor}}$ to replace MSE, following AlphaFold2 (Jumper et al., 2021). The third and final block of Table 1 shows that FAPE significantly improved structure designability TM‐score to values well above 0.7 and an RMSD lower than 2.0, but at the cost of sequence self‐consistency and structure‐to‐sequence cross‐consistency. Using a larger AFDB dataset did not alleviate this trade‐off. One contributing factor is that the magnitude of FAPE during training was larger than that of MSE in our case, causing the frame position loss $L_{\text{coor}}$ to dominate over the amino acid type loss $L_{\text{type}}$ and reducing the emphasis on sequence quality. Adjusting sw across losses as hyperparameters could help mitigate this imbalance and may be explored with additional computational resources. Nevertheless, when structure quality such as designability is prioritized, as in motif scaffolding tasks, FAPE may be a more suitable choice for the position loss than MSE. We also found that random masking made the designability TM‐score drop from 0.784 to 0.737 and RMSD increase from 1.792 to 2.157 Å (close to the 2 Å threshold that suggests high‐quality structures), but slightly improved all other metrics and likely improved sequence quality. So for a balanced view, we selected the configuration with random masking for JointDiff‐x (FAPE).

### 3.3 Evaluating consistency in monomer designs from JointDiff and competing methods

We then compared JointDiff and JointDiff‐x with state‐of‐the‐art diffusion‐based protein design methods including structure designers FoldingDiff, RFdiffusion, and Chroma as well as two‐stage protein designers including Chroma (followed by a Potts model) and ProteinGenerator. RFdiffusion + ProteinMPNN for co‐design was not considered here because ProteinMPNN is part of the evaluation pipeline. Besides self‐consistency within designed sequences or structures, cross‐consistency between designed sequences and structure pairs was also evaluated.

As discussed in Section 2.6, these metrics reflect the model's ability to learn and generate across three data modalities: sequence, frame positions, and frame orientations. Foldability captures sequence plausibility; designability assesses backbone structure quality by evaluating both frame positions and orientations; and sequence–structure cross‐consistency measures the compatibility across all three modalities. Importantly, these metrics have been shown to correlate with experimental success in protein design. A key metric is the designability or the sequence‐to‐structure consistency of less than 2 Å in RMSD.

Table 2 summarizes self‐ and cross‐consistency evaluations for designs generated by various methods. Additional density plots are in Figure S2. To validate the assessment metrics, we first examined their values on natural proteins. The average RMSD for structure designability and sequence‐to‐structure consistency was approximately 1.5 Å, below the 2 Å threshold commonly associated with experimental success. These RMSD values primarily reflect errors from structure prediction tools (e.g., AlphaFold2 or, in this case, ESMFold).

**TABLE 2:** Consistency (self and cross) and speed evaluations for designs from diffusion‐based state‐of‐the‐art methods and ours. The best performance from protein co‐designers in each metric was boldfaced. Publicly available trained models for Chroma and ProteinGenerator were used, thus their performances could be overestimated due to potential data leakage. Structure‐to‐sequence cross‐consistency (0.3) was lower than sequence self‐consistency (0.4), even for leading co‐designers, indicating the difficulty of generating sequences that match a given structure. While JointDiff achieves simultaneous sequence and structure generation within a unified framework, it shows competitive structure generation but weaker sequence performance, suggesting limitations of multinomial diffusion for modeling discrete sequence distributions. These results highlight the challenges of multimodal co‐design.

Model	Self‐consistency		Cross‐consistency		Time
	seq (fold.)	str (design.)	str2seq	seq2str	(s)
	↑	TM↑/RMSD↓	↑	TM↑/RMSD↓	↓
Nature	0.436 ± 0.11	0.859 ± 0.15/1.438 ± 0.90	0.403 ± 0.10	0.869 ± 0.16/1.450 ± 0.86	‐
Structure design only					
FoldingDiff	‐	0.368 ± 0.20/2.869 ± 1.25	‐	‐	0.99
RFdiffusion	‐	0.888 ± 0.11/1.126 ± 0.72	‐	‐	819.08
Chroma	‐	0.670 ± 0.17/2.451 ± 1.07	‐	‐	39.67
Structure design followed by structure‐conditioned sequence design					
Chroma + Potts	**0.419** ± 0.09	0.670 ± 0.17/2.451 ± 1.07	**0.327** ± 0.08	**0.769**±0.16/**2.016** ± 0.94	42.23
Iteratively sequence design followed by structure prediction and refinement					
ProteinGenerator	0.385 ± 0.12	**0.862** ± 0.13/**1.263** ± 0.65	**0.327** ± 0.09	0.738 ± 0.14/2.218 ± 1.04	68.10
Our sequence–structure joint design					
JointDiff	0.217 ± 0.05	0.692 ± 0.13/2.249 ± 1.16	0.199 ± 0.04	0.591 ± 0.15/2.724 ± 1.25	**2.58**
JointDiff‐x	0.255 ± 0.07	0.647 ± 0.13/2.589 ± 1.14	0.205 ± 0.06	0.469 ± 0.11/3.351 ± 1.29	2.64
JointDiff‐x (FAPE)	0.208 ± 0.06	0.737 ± 0.15/2.157 ± 1.02	0.166 ± 0.05	0.357 ± 0.09/3.790 ± 1.25	2.71

Abbreviation: FAPE, Frame Aligned Point Error.

Sequence foldability and structure‐to‐sequence consistency values were around 0.4 for natural proteins. These values are not necessarily low; rather, they reflect the biological reality that protein sequence–structure relationships are not one‐to‐one. Structure is generally more conserved than sequence, and many sequences—especially homologs with ≥30% sequence identity—can adopt similar backbone structures. These values therefore capture not only limitations in inverse design tools (e.g., Evolutionary Scale Modeling ‐ Inverse Folding [ESM‐IF], or in this case, ProteinMPNN) but also the inherent variability and redundancy in protein sequence–structure mappings.

For designed structures, based on RMSD as a measure of designability, JointDiff (2.249 Å) and JointDiff‐x (FAPE variant) (2.157 Å) performed comparably or better than the two‐stage Chroma (2.451 Å), though worse than ProteinGenerator (1.263 Å). Their RMSD values are close to the experimentally validated threshold of 2 Å, suggesting reasonable structural fidelity. JointDiff‐x (default MSE variant) (2.589 Å) was slightly worse. Among structure‐only generators, RFdiffusion achieved the best performance (1.126 Å), comparable to the sequence–structure codesigner ProteinGenerator.

For designed sequences, assessed via foldability, all JointDiff variants lagged behind competing methods (0.21–0.26 vs. 0.39–0.42). While JointDiff scores approached 0.3—the sequence identity threshold often used to define protein family membership—they remained below the ~0.4 values achieved by other co‐designers.

For designed sequence–structure pairs, JointDiff and JointDiff‐x showed lower cross‐consistency than the two‐stage Chroma and ProteinGenerator. Specifically, JointDiff's sequence‐to‐structure cross‐consistency was around 2.7 Å RMSD, slightly above the 2 Å threshold, whereas Chroma and ProteinGenerator achieved 2.0 and 2.2 Å, respectively. Structure‐to‐sequence cross‐consistency for JointDiff was around 0.2, below the 0.3 threshold, which was barely met by Chroma and ProteinGenerator (both ~0.33).

Taken together, these results highlight the challenge of multimodal co‐design. Structure‐to‐sequence cross‐consistency was notably worse than sequence self‐consistency (0.3 vs. 0.4) even for leading co‐designers, underscoring the difficulty of generating sequences that match a given structure. While JointDiff models and generates sequence and structure simultaneously within a unified framework, it did not improve cross‐consistency. Its structure generation was competitive with leading two‐stage co‐designers, but its sequence generation was weaker. These findings suggest a potential limitation of multinomial diffusion in modeling discrete sequence distributions.

**FIGURE 4:**
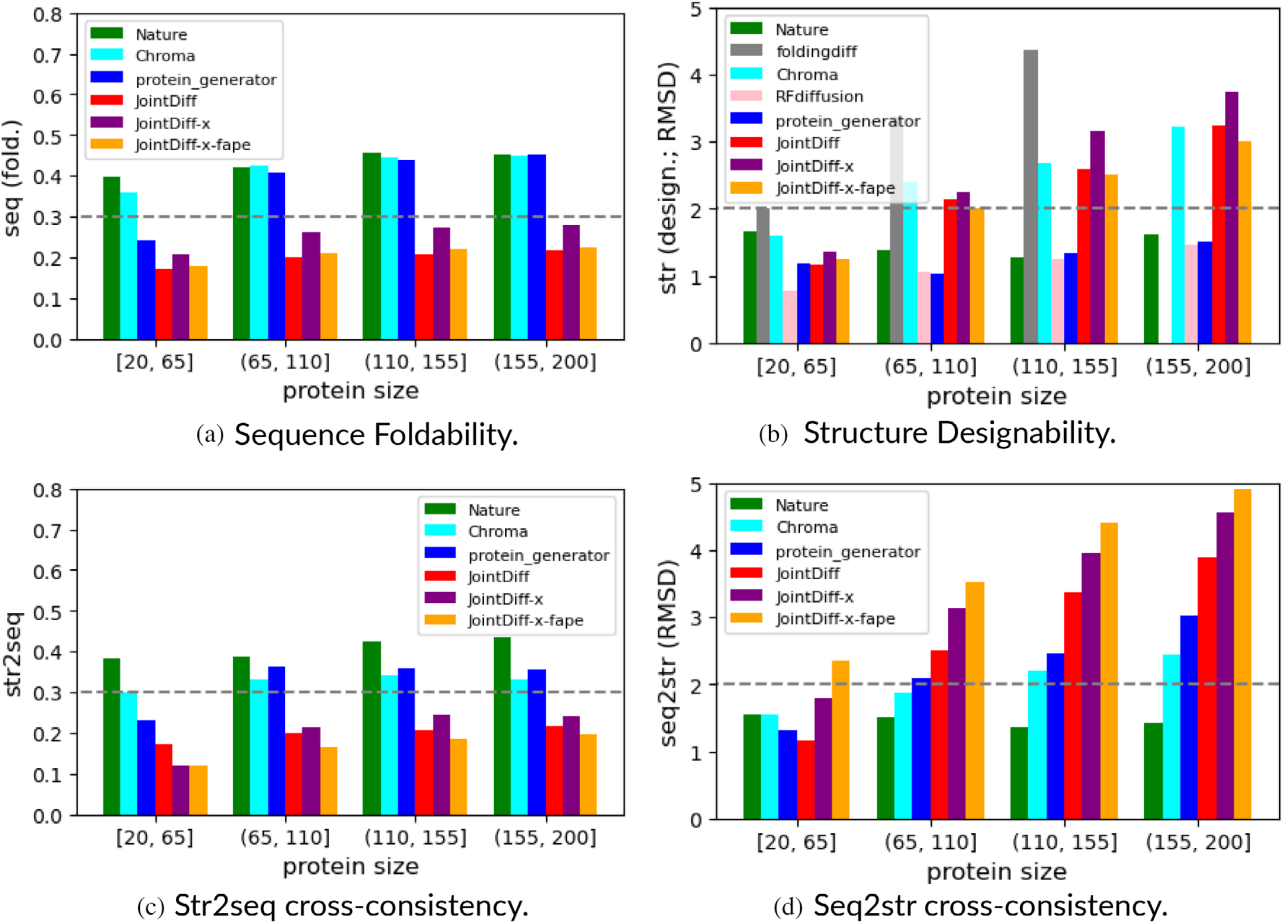
Consistency evaluations across different protein length bins. (a)–(d) show performances in four metrics across different protein size bins. For structure generation, JointDiff matched ProteinGenerator in designability for short proteins (20–65 residues) but declined with increasing size, where ProteinGenerator maintained better RMSD values. For sequence and sequence–structure co‐design, foldability and cross‐consistency metrics showed that Chroma and ProteinGenerator outperformed JointDiff across all bins, with JointDiff's limitations becoming more pronounced for longer proteins. In (b), Foldingdiff can only generate proteins with no more than 128 residues. (a) Sequence foldability. (b) Structure designability. (c) Str2seq cross‐consistency. (d) Seq2str cross‐consistency.

To examine the impact of protein length, we binned the evaluation metrics by protein length, as shown in Figure 4. For structure generation, JointDiff matched the leading ProteinGenerator in designability for small proteins (length 20–65), but its performance declined for larger proteins, where ProteinGenerator maintained superior RMSD values. For sequence generation, foldability scores for both co‐designers, Chroma and ProteinGenerator, were relatively insensitive to protein length and consistently outperformed JointDiff across all bins. For sequence–structure pair generation, the limitations of JointDiff's sequence modeling and the size‐dependent decline in structure quality were reflected in the cross‐consistency metrics. Specifically, sequence‐to‐structure consistency worsened with increasing protein length, and structure‐to‐sequence consistency remained low across all bins, reinforcing the challenges of simultaneous co‐design in longer proteins.

Table 2 also showed that JointDiff generates an average protein pair every 2.5 s, which is 16 and 26 times faster than the two‐stage Chroma (42 s) and ProteinGenerator (68 s), respectively. JointDiff is also two orders of magnitude faster than the structure generator RFdiffusion (819 s on average). We followed the official RFdiffusion implementation for inference under the default configuration of 50 time steps, with setup time excluded from the measurement. All inference time was evaluated using a batch size of 1 on a Tesla T4 GPU (16 G).

### 3.4 Evaluating diversity, novelty, and sanity checks in monomer designs

**FIGURE 5:**
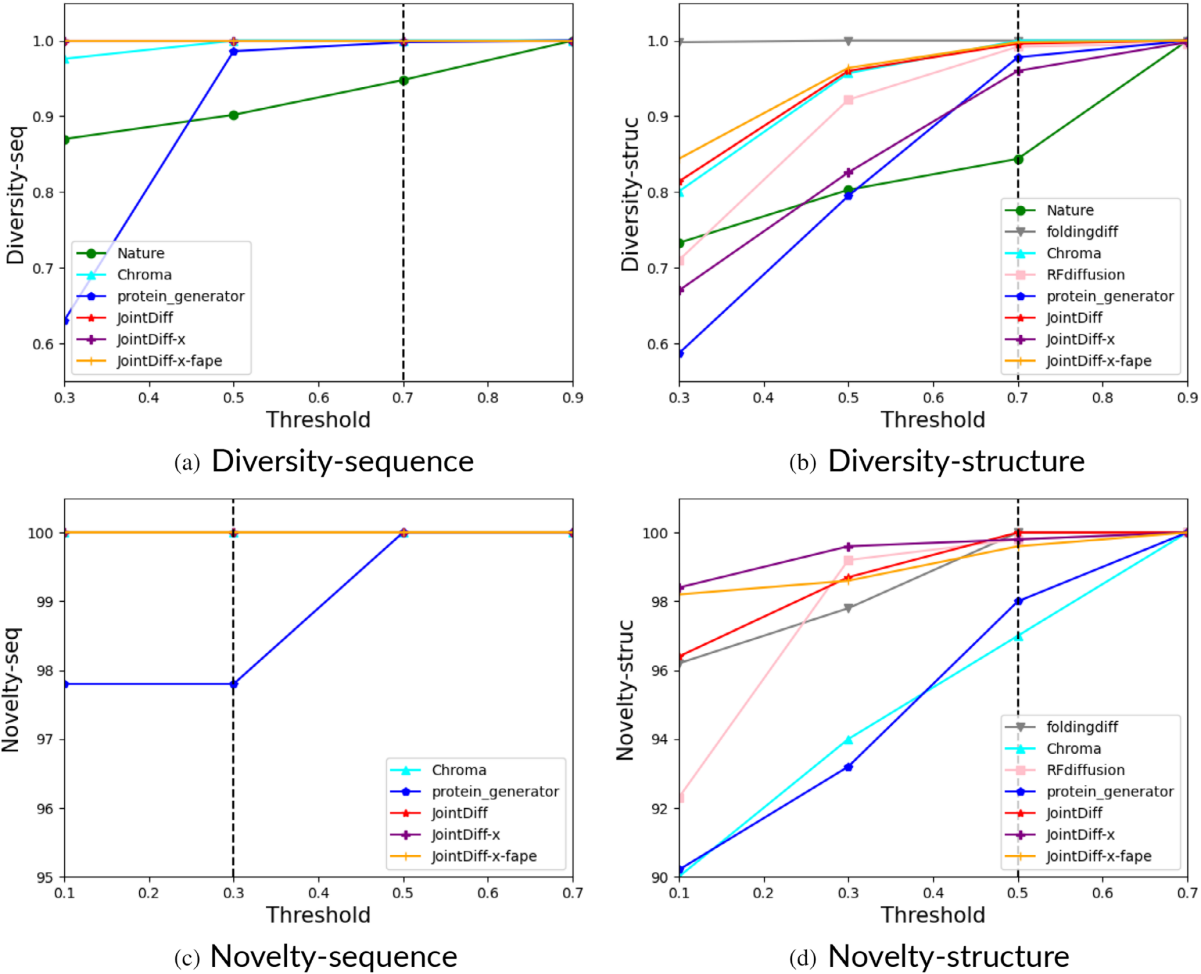
Diversity and novelty curves under varying sequence and structure similarity thresholds. Default thresholds (0.7 for sequence/structure diversity, 0.3 for sequence novelty, and 0.5 for structure novelty) are marked by vertical dashed lines. At a stricter sequence identity threshold of 0.3, ProteinGenerator's diversity dropped sharply to 64%, compared to 88% for natural proteins and nearly 100% for diffusion‐based models. For structure diversity at a TM‐score threshold of 0.5, FoldingDiff maintained nearly 100%, while other models ranged between 80% and 95%, and sequence/structure novelty remained consistently high across all models. (a) Diversity‐sequence. (b) Diversity‐structure. (c) Novelty‐sequence. (d) Novelty‐structure.

Besides self‐ and cross‐consistency evaluations, we evaluated sequence or structure diversity and novelty to assess how well each model explores new regions of protein space. While Table S3 shows that all diffusion‐based models achieve nearly 100% diversity and novelty under default thresholds, we further investigated whether these metrics remain meaningful under stricter criteria. To this end, we varied the similarity thresholds for both sequence and structure clustering and plotted the resulting diversity and novelty curves in Figure 5. The default thresholds—0.7 in sequence identity or TM‐score for diversity, 0.3 in identity for sequence novelty, and 0.5 in TM‐score for structure novelty—are indicated by vertical dashed lines.

For sequence diversity, when the identity threshold was reduced to 0.3—a level typically used to define homologous sequences—diversity dropped sharply to 64% for ProteinGenerator but remained close to 100% for all other diffusion‐based models. For comparison, natural proteins showed 88% diversity at this threshold. For structure diversity, when the TM‐score threshold was reduced to 0.5—a level corresponding to the same structural fold—FoldingDiff maintained nearly 100% diversity, while other models dropped to between 80% (ProteinGenerator, comparable to natural proteins) and 95% (RFdiffusion, Chroma, and our JointDiff). In contrast, sequence and structure novelty remained high across all models, with only slight decreases under stricter thresholds.

Table S3 also shows that the generative designs passed sanity checks: extremely low amino acid repeats (5‐mer or longer) and low pairwise residue clashes. JointDiff exhibited a relatively higher clash count (30.26), though still acceptable (0.3% of all residue pairs for an average 100‐residue protein). This count was found to depend on the sw used during preprocessing: smaller sw values, leading to compressed structures prior to diffusion, increased clashes in the rescaled outputs (Table S2, centered structures). This effect may be attributed to the unit Gaussian prior and noise prediction used in DDPM. In contrast, JointDiff‐x reconstructs ground‐truth coordinates rather than noises and incorporates additional structure‐aware losses (e.g., distance, distogram, and clash), resulting in a significantly reduced clash count (8.13), comparable to FoldingDiff (7.86).

### 3.5 Evaluating speed

**FIGURE 6:**
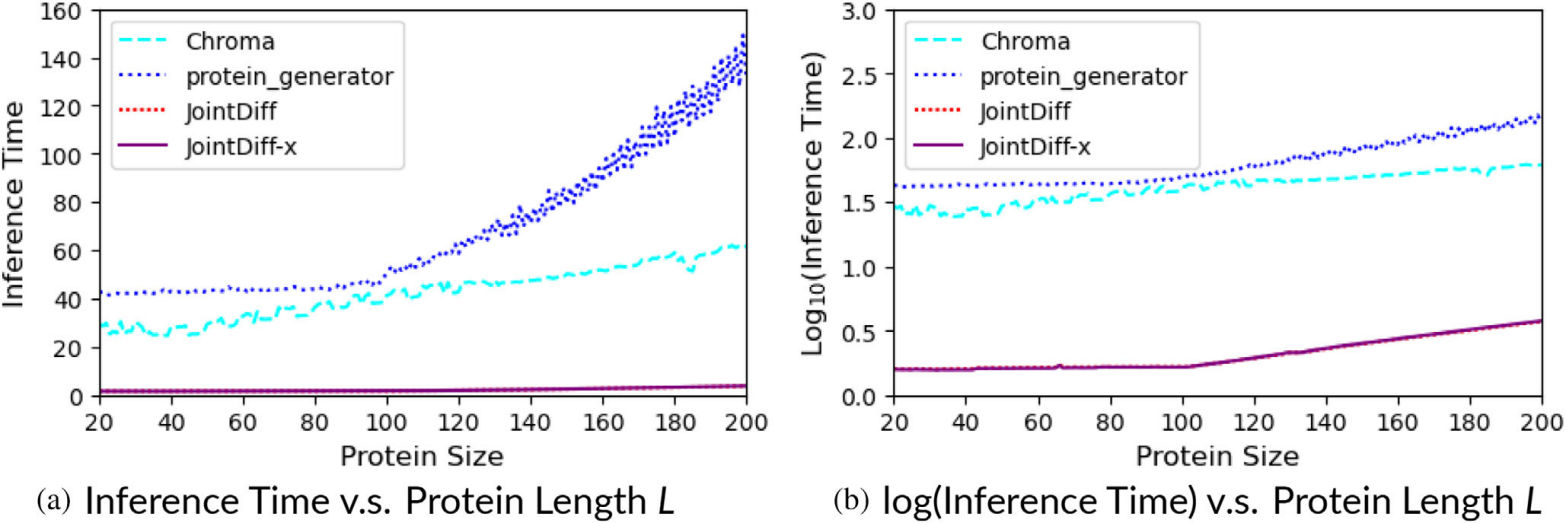
Design speed as a function of protein length, with all models benchmarked on a single Tesla T4 (16 GB) GPU. ProteinGenerator was the slowest, showing exponentially increasing inference times beyond 100 residues, while Chroma scaled approximately linearly with length. In comparison, JointDiff and JointDiff‐x were the fastest (1.5–3 vs. 25–60 s for Chroma and 40–150 s for ProteinGenerator), with their speed advantage growing at longer lengths; JointDiff‐x (Frame Aligned Point Error) was excluded as it shares the same architecture and inference pipeline as JointDiff. (a) Inference time versus protein length *L*. (b) log(inference time) versus protein length *L*.

Table 2 showed earlier that JointDiff generates an average protein pair 16‐ and 26‐times faster than the two‐stage Chroma and ProteinGenerator, respectively. We next examine their design speed in relationship to protein size (Figure 6). All model inference jobs were run on a single Tesla T4 (16 GB) GPU for fair comparison. ProteinGenerator was the slowest co‐designer among the three and its inference time grew exponentially as the protein length increased, with a significantly steeper increase as the length went above 100. Chroma was faster with inference time approximately linear in design length. Our JointDiff and JointDiff‐x (default MSE variant) were the fastest (1.5–3 vs. 25–60 s for Chroma and 40–150 s for ProteinGenerator). Their speed advantage increased, especially compared to ProteinGenerator, when the design length increased. JointDiff‐x (FAPE) was not included in the analysis since it has exactly the same architecture and inference pipeline as the default JointDiff‐x.

### 3.6 Classifier‐guided sampling efficiency

Given that JointDiff models are 1–2 orders of magnitude faster than competing methods, a scenario where they could excel is rapid iteration under external feedback, computational or experimental, toward desired properties. In short, rapid generation enables iterative design cycles. To illustrate this, we evaluated classifier‐guided sampling over 100 time steps with JointDiff‐x and compared its performance to RFdiffusion under identical conditions on a single A100 GPU (40 GB).

As described in Section 2, a pretrained CATH classifier from Chroma was used to guide generation toward specific structural architectures. We selected four benchmark architectures with high classifier accuracy: 1.10 (orthogonal bundle), 2.60 (sandwich), 3.30 (two‐layer sandwich), and 3.40 (three‐layer [aba] sandwich).

**FIGURE 7:**
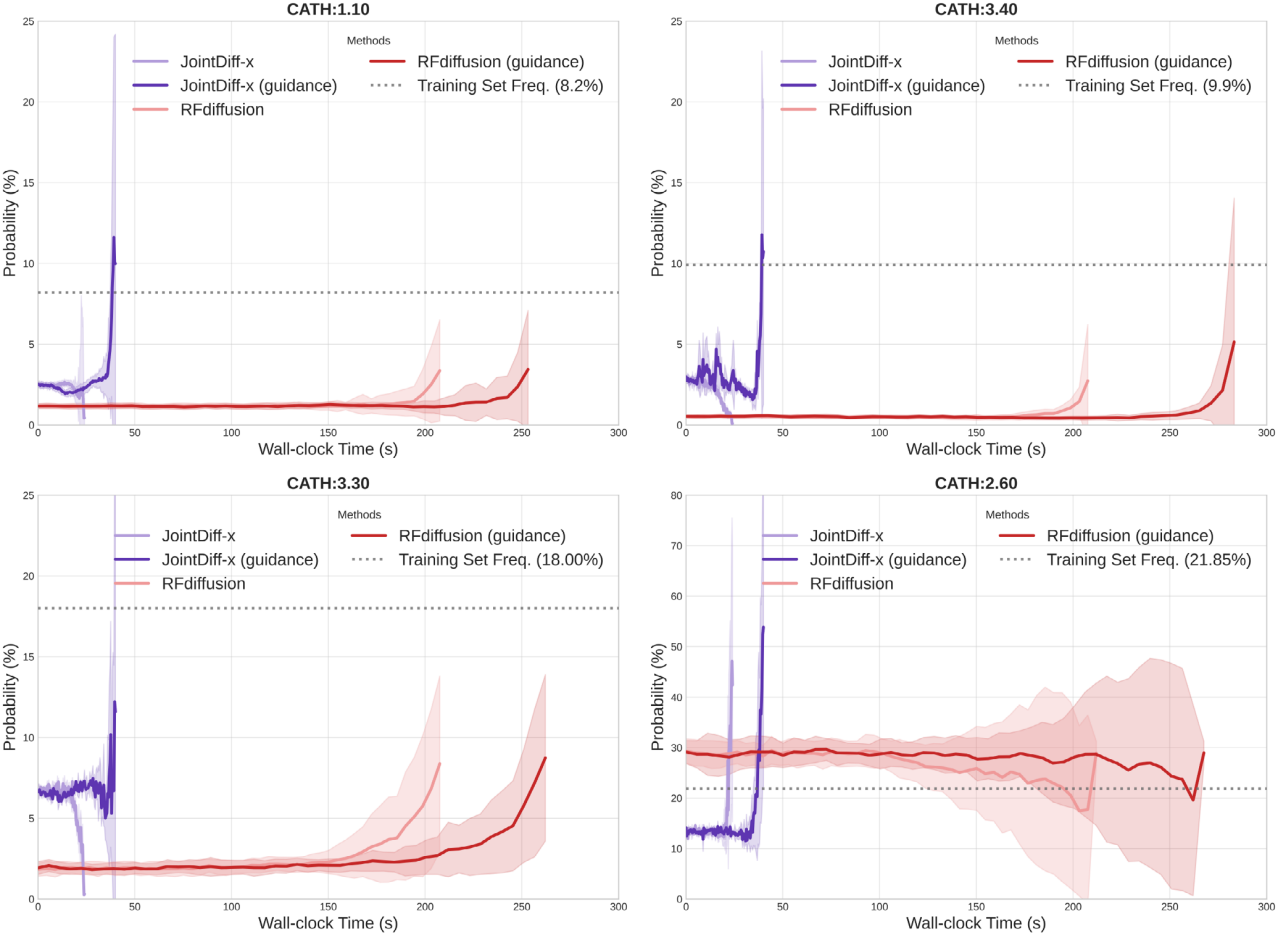
Probability of achieving the target architecture versus wall‐clock time, comparing unguided and classifier‐guided sampling. Each panel corresponds to one of the four target CATH architectures: (top left) 1.10, (top right) 3.40, (bottom left) 3.30, and (bottom right) 2.60. Solid lines indicate the mean classifier‐assigned probability of the target architecture across 40 sample trajectories; shaded regions denote variability. Although for both JointDiff‐x and RFdiffusion, guided versions (darker lines) take longer in each inference step than unguided ones (lighter lines) due to additional steering during generation, they often achieve higher success rates toward the end. JointDiff‐x with guidance rapidly attains high target‐architecture probability within tens of seconds, while RFdiffusion requires substantially longer runtimes and generally fails to achieve comparable probability levels within the plotted horizon.

Figure 7 shows the probability trajectories for each target architecture. Across all cases, JointDiff‐x with classifier guidance exhibits a markedly faster rise in target‐class probability compared to RFdiffusion. For architectures 3.40, 3.30, and 1.10, JointDiff‐x reaches meaningful probability levels—comparable to the dotted reference lines indicating training frequencies—within approximately 30–45 s. In contrast, RFdiffusion remains near baseline for most of the runtime and only begins to increase substantially after 200 s. For architecture 2.60, JointDiff‐x achieves probabilities exceeding 50% within the first ~40 s, while RFdiffusion plateaus between 25% and 30% and does not reach comparable peaks. For completeness, we note that runtime differences compared to Table 2 reflect variations in experimental settings. JointDiff‐x without classifier guidance is slower here (over 20 vs. 2.5 s previously), primarily because this version includes additional fold‐probability computations (18.75 s on average for 100 time steps). Conversely, RFdiffusion without guidance is faster here (just over 200 s vs. an average of over 800 s across all lengths previously), likely due to shorter protein lengths and the use of a higher‐performance GPU.

These results demonstrate that classifier guidance applied directly to the clean C_α_ predictions in JointDiff‐x yields rapid and stable improvements. RFdiffusion, despite using the same classifier signal and decay schedule, converges more slowly and incurs higher wall‐clock costs. Whether measured by time‐to‐threshold (time to reach a given probability level) or area under the probability–time curve within a fixed budget, JointDiff‐x consistently outperforms RFdiffusion in guided design efficiency.

### 3.7 Motif scaffolding with inpainting‐enabled diffusion

The preceding results focused on unconditional monomer design. To explore more application‐relevant tasks, we evaluated our model on motif scaffolding. The random masking (inpainting) strategy used during training, originally intended to improve unconditional monomer design, also enables conditional motif scaffolding. Although this strategy did not significantly improve monomer design performance in earlier results, it proved beneficial for motif scaffolding, particularly when combined with the positional diffusion loss $L_{\text{coor}}$ in FAPE rather than MSE.

To assess the effectiveness of our approach, we followed the RFdiffusion benchmark and evaluated our models on 21 single‐chain cases from the original 24‐task motif scaffolding benchmark. We used the same evaluation metrics as in monomer design, focusing on designability defined by RMSD below 2 Å as the in silico success criterion, consistent with RFdiffusion. We compared our models against two state‐of‐the‐art structure‐only scaffolding methods: RFdiffusion (Watson et al., 2023) and FrameFlow (Yim et al., 2024). Results are summarized in Table 3.

**TABLE 3:** Self‐consistencies and cross‐consistencies on 21 motif scaffolding benchmark tasks from RFdiffusion. “MF” refers to motif factor in training loss. We still evaluated the models with self‐consistency (sequence foldability and designability). For the baselines we calculated the sequence foldability of the sequences from ProteinMPNN. Besides we also calculated the success rate following RFdiffusion and the benchmark score from (Zheng et al., 2025). For the co‐design models, we reported the performance on the cross‐consistency as well. JointDiff‐x‐mse (*w*/*o*) is the default JointDiff‐x for monomer design and JointDiff‐x‐mse (MF = 0.0) refers to JointDiff‐x with random masking. Boldfaced configurations were adopted for JointDiff‐x and boldfaced entries highlight the top results for each evaluation metric. A higher value is preferred for all metrics except RMSD.

Model	MF	Self‐consistency ↑				Cross‐consistency ↑	
		seq (fold.)	str (design.)	succ. rate (%)	succ. rate (%)	str2seq	seq2str
			TM/RMSD	(TM ≥0.7)	(RMSD ≤2.0)		TM/RMSD
RFdiffusion		‐	**0.792**/**1.496**	**82.2**	**89.6**	‐	‐
FrameFlow		‐	0.736/1.998	77.8	75.9	‐	‐
JointDiff	0.0	0.228	0.449/4.177	17.1	14.9	0.201	0.374/3.887
JointDiff‐x (MSE)	w/o	**0.275**	0.290/4.314	4.4	3.3	0.160	0.337/4.755
	0.0	0.248	0.320/4.400	2.6	2.4	0.181	0.313/4.281
	1.0	0.264	0.435/3.806	4.9	17.5	0.204	0.351/3.991
	10.0	0.250	0.276/4.641	4.2	3.0	0.146	0.286/4.570
**JointDiff‐x (FAPE)**	0.0	0.245	0.556/3.377	34.7	28.6	**0.220**	0.328/**3.850**
	**1.0**	0.219	0.665/2.965	64.0	54.9	0.200	**0.469**/3.880
	1.0 (AFDB)	0.207	0.635/3.105	62.4	52.9	0.196	0.380/4.132
	10.0	0.222	0.635/2.999	59.8	54.3	0.198	0.371/4.278

Abbreviations: AFDB, AlphaFold Protein Structure Database; FAPE, Frame Aligned Point Error; MSE, Mean Squared Error.

Compared to monomer design, JointDiff and JointDiff‐x with MSE‐based position loss showed substantially lower performance on motif scaffolding, with average designability RMSD around 4 Å—significantly worse than competing models (1.5–2.0 Å). Using the RMSD <2 Å threshold, the success rate for JointDiff‐x (MSE) without random masking—the same configuration used in monomer design—was only 3.3%. In contrast, RFdiffusion and FrameFlow achieved success rates of 89.6% and 75.9%, respectively. Incorporating random masking improved performance only when the motif factor was set to 1 (rather than 0 or 10, the latter being the default in RFdiffusion), but the success rate remained modest at 17.5%.

However, incorporating FAPE loss led to a marked improvement for JointDiff‐x: the average designability RMSD improved to around 3 Å, and the success rate increased to 54.9%. This improvement was not observed in monomer design, suggesting that motif scaffolding is more sensitive to SE(3)‐aware supervision. Since motif residues are fixed in space, even small rotational or translational deviations can introduce structural gaps. FAPE, by aligning local frames during training, helps the model better preserve these spatial constraints.

While our overall performance remains modest compared to the best structure‐only models, these results demonstrate the potential of our joint sequence–structure diffusion framework for functional site scaffolding and highlight the importance of SE(3)‐aware losses in such tasks. To further explore this potential in a biologically meaningful context, we next applied the JointDiff‐x FAPE model trained with random masking to the design of GFPs, using fixed chromophore motifs as scaffolding constraints.

### 3.8 Computational design and experimental validation of GFPs

Building on the motif scaffolding capabilities of our model, we conducted a case study on GFPs, a well‐characterized protein family with clear structural and functional motifs. This experiment served as both a functional validation of our model and a test of its ability to generate diverse, plausible designs under biologically relevant constraints. The initial round prioritizes diversity and functional motif preservation over brightness optimization.

Following the design and filtering process described in Section 2.8, we obtained a final set of 32 candidate GFP designs. Reliable sequencing data could not be obtained for seven of these designed variants, leaving 25 for further analysis. Based on sequence identity to the wild‐type GFP templates, the 25 variants were grouped into three categories: Low (*L*, <50% identity), Medium (*M*, 50%–70%), and High (*H*, ≥70%), with 9, 8, and 8 in these groups, respectively.

We experimentally tested the 25 variants' fluorescence activity in *E. coli*. DNA sequences were cloned into the pBbB8k expression vector under the strong BBa_J23119 constitutive promoter and transformed into *E. coli* NEB 5‐alpha. After plasmid extraction and sequencing, 16 variants (6 Low, 7 Medium, and 3 High) were advanced for fluorescence quantification; the remaining nine were excluded due to sequence mutations.

**FIGURE 8:**
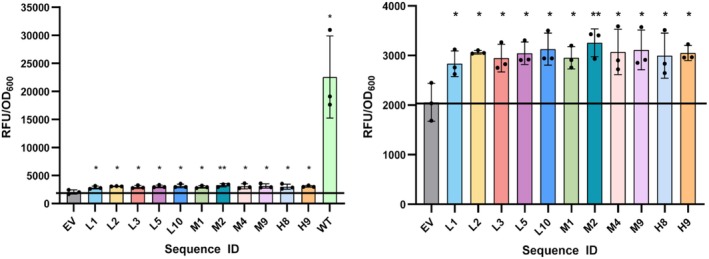
Left: Fluorescence intensity of *Escherichia coli* whole‐cells expressing the designed green fluorescent protein (GFP) variants 48 h after inoculation. *E. coli* cells expressing avGFP (wild type [WT]) served as the positive control, and cells harboring a plasmid without the GFP gene (empty vector [EV]) was used as a negative control. Right: A zoomed‐in view of the data presented in the left, showing the relative fluorescence of each design variant when compared to the EV. Experiments were conducted in triplicate, and data shown are mean values (±standard deviation). Statistical significance was evaluated using unpaired Student's *t*‐tests. *p < 0.05; **p < 0.01. OD, optical density; RFU, relative fluorescence intensity.

The final 16 variants were cultured for 24 and 48 h, and whole‐cell fluorescence was measured to assess GFP expression. Since wild‐type avGFP showed higher fluorescence at 48 h (Figures 8 and S3), this timepoint was used for screening. Fluorescence levels were compared against avGFP (positive control) and an EV (negative control).

Overall, 11 variants of the 16 (Figure 8) exhibited significantly higher fluorescence than the EV control. Their sequence and structural similarities to known templates, along with fluorescence intensities, are summarized in Table 4. Notably, all 11 designed structures adopted beta‐barrel folds closely resembling the templates, with TM‐scores ranging from 0.93 to 0.98, suggesting similar functional activity despite less than 10% of the template structure being provided as the conditioning motif (residues 58–71 on a central helix and highly conserved residues 96 and 222). The top‐performing design, variant M2, showed significantly higher fluorescence (*p* < 0.01) than EV and was 17.2‐fold less bright than avGFP. M2 shared 59.8% sequence identity with its template (PDB: 1QY3). Importantly, variant L3, with the lowest sequence identity (39.7% identity to the template wild‐type avGFP [PDB: 1QY3]; 39.7% to the closest blastP (Altschul et al., 1997; Altschul et al., 2005) hit in the non‐redundant clustered database [Accession: XAJ63113.1]), also showed significant fluorescence (*p* < 0.01), at 23‐fold lower brightness than avGFP.

**TABLE 4:** Evaluation of green fluorescent proteins (GFP) designs generated by JointDiff‐x motif scaffolding: sequence identity to template GFP structures and the ClusteredNR database (BLASTp), structural similarity to template GFP structures, and fold‐change in fluorescence intensity relative to avGFP. In one round of design, 11 out of 16 valid variants were found with measurable fluorescence with statistical significance, including L3 with less than 40% sequence identity to known GFPs. Boldfaced entries highlight the top results for each evaluation metric.

ID	Template PDB	Seq. similarity (%) ↓ to template/database	Str. similarity (TM) ↑ to template	Fold‐difference ↓ in fluorescence intensity
L1	5B61	48.8/42.4	0.96	26.3
L2	5B61	48.9/43.5	0.96	20.3
L3	1QY3	**39.7**/39.7	0.93	23.0
L5	5B61	43.7/**37.5**	0.95	20.8
L10	2B3P	44.4/44.4	0.94	19.1
M1	1QY3	66.8/67.0	0.96	22.8
M2	1QY3	59.8/59.8	0.96	**17.1**
M4	1QY3	65.5/66.4	0.95	20.2
M9	1QY3	60.7/61.4	0.96	19.4
H8	5B61	74.2/68.1	**0.98**	21.8
H9	5B61	76.0/69.9	**0.98**	20.6

These results demonstrate that our model can generate evolutionarily distant GFP variants with measurable fluorescence, establishing a promising starting point for further optimization. While the top‐performing variant (M2) was 17.2‐fold less bright than avGFP, it still exhibited significant fluorescence despite sharing only 59.8% sequence identity with its template. Notably, variant L3, with just 39.7% identity to the closest GFP, retained measurable activity, suggesting functional preservation.

A recent study using the ESM‐3 generative language model reported results from their initial round of GFP design (Experiment 1), conducted prior to focused optimization. In that round, approximately 17 out of 88 designs exhibited measurable fluorescence. Among them, a variant (B8) with 36% sequence identity to avGFP and 57% to tagRFP was approximately 50‐fold less bright than natural GFPs (Hayes et al., 2025), yet was selected for the subsequent round of focused optimization (Experiment 2). While experimental protocols differ and direct comparisons are not appropriate, these results provide useful context for interpreting our own first‐round designs, which similarly prioritize diversity and functional motif preservation over brightness optimization. Both studies demonstrate the feasibility of designing functional, low‐homology GFP variants. Our results thus establish a strong baseline for future rounds of focused design and optimization for even brighter GFPs, similar in spirit to the iterative improvements seen in ESM3 workflows.

## 4 DISCUSSION

In this study, we introduced multimodal diffusion models, JointDiff and JointDiff‐x, to learn the joint distribution of protein sequence–structure pairs. Compared to popular two‐stage approaches that generate one modality first and then conditionally generate the other, our models simultaneously generate protein sequence and structure in a unified framework, which is hypothesized to enhance cross‐modality interactions and promote coherent, functional designs. Through comprehensive evaluations of sequence or structure self‐consistency and sequence–structure cross‐consistency, we found that JointDiff models achieved similar or better monomer structure designability on average compared to leading two‐stage methods Chroma and ProteinGenerator. Their structure designability decreases, however, for larger proteins. JointDiff generates protein designs an order of magnitude faster, facilitating efficient design–test loops for protein discovery, as demonstrated in accelerated design for desired structural architectures through guided sampling without retraining.

While our models demonstrate competitive monomer structure quality and excel in generation speed, enabling efficient iterative design cycles, they currently fall short in monomer sequence quality and motif scaffolding performance. In particular, designed sequences showed room for improvement, as reflected in sequence self‐consistency and structure‐to‐sequence cross‐consistency metrics. This limitation is possibly due to the fact that amino acid types are discrete and modeled as a multinomial distribution, which poses challenges for diffusion models originally developed for continuous domains such as images and point clouds. Moving forward, algorithmic advances in multimodal alignment and generative modeling are needed to improve functional protein co‐design. Multimodal language models such as ESM3 represent promising directions by tokenizing structural features. Interestingly, ESM3 does not generate all modalities simultaneously and still opts for sequential generation across modalities.

To further validate our model in a biologically relevant context, we conducted a case study to computationally design and experimentally test GFPs. Several evolutionarily distant variants generated by our model exhibited measurable fluorescence in *E. coli*, despite the low sequence similarity to known GFPs. These results demonstrate the model's ability to scaffold functional motifs and generate viable candidates for experimental testing. While fluorescence levels were lower than wild‐type GFP, they are comparable to first‐round designs from other generative models such as ESM3. This establishes a strong baseline for future rounds of optimization and highlights the potential of multimodal diffusion frameworks in functional protein design.

Notably, our experimental results indicate that several sequences generated by JointDiff‐x motif scaffolding exhibited functional activity, even when computational metrics suggested limited sequence quality. This highlights the importance of empirical validation in complementing in silico assessments, especially when structural criteria alone are often used to evaluate motif scaffolding success in silico.

Due to computational resource limitations, our models were trained on CATH proteins with no more than 200 residues or AFDB proteins with no more than 300 residues. The models' memory usage on Tesla T4 GPUs was around 3, 4, and 11.3 GB when trained with proteins of length 100, 200, and 300, respectively, which led to out‐of‐memory (OOM) issues for larger proteins. Nevertheless, models trained on smaller proteins are still capable of performing inference on larger ones. The OOM issue can also be mitigated using techniques such as cropping. In future work, we anticipate that performance can be further enhanced by leveraging larger models, expanded databases, and more diverse training samples.

Our findings underscore the promise of unified multimodal frameworks for accelerating functional protein design. Future work will explore scaling to larger proteins as well as extending to multi‐chain complexes and context‐aware binder design, such as partner‐specific binder generation and antigen‐specific antibody complementarity‐determining region (CDR) design.

## AUTHOR CONTRIBUTIONS

**Shaowen Zhu:** Writing – original draft; data curation; software; visualization; methodology; validation; writing – review and editing; investigation. **Siddhant Gulati:** Investigation; methodology; writing – review and editing; writing – original draft; data curation. **Yuxuan Liu:** Investigation; writing – original draft; methodology; validation; visualization; writing – review and editing; software; data curation. **Siddhi Kotnis:** Data curation; investigation; validation. **Qing Sun:** Supervision; resources; project administration; validation; visualization; writing – review and editing; methodology; investigation; funding acquisition; writing – original draft. **Yang Shen:** Conceptualization; writing – review and editing; project administration; supervision; resources; formal analysis; methodology; validation; visualization; funding acquisition; investigation.

## CONFLICT OF INTEREST STATEMENT

The authors declare no conflicts of interest.

## Supporting information


**Data S1.** Supporting Information.

## Data Availability

The data that support the findings of this study are openly available in GitHub at https://github.com/shen-Lab/JointDiff.
