# Supplementary material for: Multimodal diffusion for joint design of protein sequence and structure
Source: Protein Sci. 2025 Nov 14;34(12):e70340. doi: 10.1002/pro.70340 (PMC12617271; doi:10.1002/pro.70340)
Supplement: Supplementary file 1 — Data S1. Supporting Information. [file PRO-34-e70340-s001.pdf]

ORIGINAL ARTICLE

Supplementary Material for "Multimodal Diffusion for Joint Design of Protein Sequence and Structure"

Shaowen Zhu[1] | Siddhant Gulati[2] | Yuxuan Liu[1] | Siddhi Kotnis[2] | Qing Sun[2,3] | Yang Shen[1*]

[1]Department of Electrical and Computer Engineering, Texas A&M University, College Station, Texas, 77843, United States

[2]Department of Chemical Engineering, Texas A&M University, College Station TX 77843, United States

[3]Interdisciplinary Graduate Program in Genetics and Genomics, Texas A&M University, College Station, TX, 77843, United States

Correspondence
Email: yshen@tamu.edu

Funding information
National Institute of General Medical Sciences, Grant/Award Number: R35GM12952;
National Institute of Allergy and Infectious Diseases, Grant/Award Number: R01AI165433;
National Science Foundation, Grant/Award Number: 1943008, 2514387, 2203715.

The supplementary material includes descriptions for modality-specific diffusion processes, algorithm details for JointDiff and JointDiff-x, as well as supplemental results on data structure distribution, computational assessments and experimental validations.

S1 | DETAILS FOR MODALITY-SPECIFIC DIFFUSION PROCESSES

S1.1 | Multinomial Diffusion for Protein Sequence

In the forward process, a noise sampled from the discrete uniform distribution was added to the one-hot encoding sequence though a weighted sum:

$$q(\boldsymbol{s}_j^t|\boldsymbol{s}_j^{t-1}) = \text{Multinomial}((1-\beta_{\text{type}}^t)\cdot \text{onehot}(\boldsymbol{s}_j^{t-1}) + \beta_{\text{type}}^t \cdot \frac{1}{20}\cdot \mathbf{1}), \tag{1}$$

where the function onehot(·) converts the amino acid type into a 20-dimensional vector, β_{type}^t is the probability of uniformly resampling amino acid over the 20 types, and **1** is the all-one vector. When $t \rightarrow T$, β_{type}^t is set close to 1 and then the noised distribution would converge to a uniform distribution. Following [1] we applied an efficient way to directly perturb $\boldsymbol{S}^0$ to $\boldsymbol{S}^t$ in the training process:

$$q(\boldsymbol{s}_j^t|\boldsymbol{s}_j^0) = \text{Multinomial}\left(\bar{\alpha}_{\text{type}}^t \cdot \text{onehot}(\boldsymbol{s}_j^0) + (1-\bar{\alpha}_{\text{type}}^t)\cdot \frac{1}{20}\cdot \mathbf{1}\right), \tag{2}$$

where $\bar{\alpha}_{\text{type}}^t = \prod_{\tau=1}^t (1-\beta_{\text{type}}^\tau)$. In the reverse process, we applied a neural network $\mathcal{F}(\cdot)$ to directly predicted position-wise multinomial distribution at step $t-1$ given the complete protein state (including sequence and structure) at step t. $\mathcal{F}(\cdot)$ included a common graph attention encoder (GAEncoder) among all three modalities (see Supplemental Sec. S2.1 for details) and a dedicated projector to amino-acid types. The transition process is written as:

$$p(\boldsymbol{s}_j^{t-1}|\boldsymbol{S}^t, \boldsymbol{X}_{C_\alpha}^t, \boldsymbol{O}^t) = \text{Multinomial}\left(\mathcal{F}(\boldsymbol{S}^t, \boldsymbol{X}_{C_\alpha}^t, \boldsymbol{O}^t)[j]\right). \tag{3}$$

Notice that the diffusions of the other two modalities of protein structure data, positions and orientations, are coupled with amino-acid type diffusion here.

S1.2 | DDPM for Frame Positions

In the forward process a random noise is added in each step such that $q(\boldsymbol{x}_j^t|\boldsymbol{x}_j^{t-1}) = \mathcal{N}(\sqrt{1-\beta_{\text{coor}}^t}\cdot \boldsymbol{x}_j^{t-1}, \beta_{\text{coor}}^t \boldsymbol{I})$. It can also been written as $\boldsymbol{x}_t = \sqrt{1-\beta_{\text{coor}}^t}\cdot \boldsymbol{x}_{t-1} + \beta_{\text{coor}}^t \cdot \boldsymbol{\epsilon}$ for implementation, while $\epsilon \sim \mathcal{N}(0,1)$, and β_{coor}^t is the diffusion rate which increases from 0 to 1 along the time step t. Following [2] we directly noised the original sample x_0 with $q(\boldsymbol{x}_j^t|\boldsymbol{x}_j^0) = \mathcal{N}(\sqrt{\bar{\alpha}_{\text{coor}}^t}\cdot \boldsymbol{x}_j^0, (1-\bar{\alpha}_{\text{coor}}^t)\boldsymbol{I})$ in the training process for efficiency, where $\bar{\alpha}_{\text{coor}}^t = \prod_{\tau=1}^t (1-\beta_{\text{coor}}^\tau)$.

For the reverse process we used a neural network estimator $\mathcal{G}(\cdot)$ (again a shared graph attention encoder (GAEncoder) across 3 modalities and a dedicated projector to coordinates) to predict the unscaled standard Gaussian noise $\boldsymbol{\epsilon} \sim \mathcal{N}(\mathbf{0}, \boldsymbol{I})$ added in the forward process such that

$\boldsymbol{x}_j^t = \sqrt{\bar{\alpha}_{\text{coor}}^t} \cdot \boldsymbol{x}_j^0 + (1 - \bar{\alpha}_{\text{coor}}^t)\epsilon_j$. Therefore the reverse process can be written as following:

$$\begin{aligned} p(\boldsymbol{x}_j^{t-1}|\boldsymbol{S}^t, \boldsymbol{X}_{C_\alpha}^t, \boldsymbol{O}^t) &= \mathcal{N}(\boldsymbol{x}_j^{t-1}|\boldsymbol{\mu}(\boldsymbol{S}^t, \boldsymbol{X}_{C_\alpha}^t, \boldsymbol{O}^t), \beta_{\text{coor}}^t \boldsymbol{I}), \\ \boldsymbol{\mu}(\boldsymbol{S}^t, \boldsymbol{X}_{C_\alpha}^t, \boldsymbol{O}^t) &= \frac{1}{\sqrt{\alpha_{\text{coor}}^t}}(\boldsymbol{x}_j^t - \frac{\beta_{\text{coor}}^t}{\sqrt{1-\alpha_{\text{coor}}^t}} \mathcal{G}(\boldsymbol{S}^t, \boldsymbol{X}_{C_\alpha}^t, \boldsymbol{O}^t)[j]). \end{aligned} \tag{4}$$

Note that the projector here also takes orientation at time $t-1$ as input to maintain SO(3)-equivariance.

S1.3 | SO(3) Diffusion for Frame Orientations

Following [3] we represent the orientations with SO(3) elements and adopt SO(3)-diffusion with the isotropic Gaussian distribution to perturb the orientations as Eq. 5 shows.

$$q(\boldsymbol{O}_j^t|\boldsymbol{O}_j^0) = \mathcal{IG}_{SO(3)}(\boldsymbol{O}_j^t|\text{ScaleRot}(\beta_{\text{orient}}^t, \boldsymbol{O}_j^0), 1 - \beta_{\text{orient}}^t). \tag{5}$$

$\mathcal{IG}_{SO(3)}(\cdot)$ refers to the isotropic Gaussian distribution and ScaleRot$(\cdot)$ would scale the rotation angles with the rotation axis fixed so as to update the rotation matrix [4]. $\bar{\alpha}_{\text{orient}}^t = \prod_{\tau=1}^t (1 - \beta_{\text{orient}}^\tau)$ where β_{orient}^t is the variance which increases with the time step.

To restore the distribution in the reverse process we used a neural network $\mathcal{H}(\cdot)$ (again a shared graph attention encoder (GAEncoder) across 3 modalities and a dedicated projector to orientations) to predict the mean orientation at step $t-1$ given the complete state (including sequence and structure) from step t:

$$p(\boldsymbol{O}_j^{t-1}|\boldsymbol{S}^t, \boldsymbol{X}_{C_\alpha}^t, \boldsymbol{O}^t) = \mathcal{IG}_{SO(3)}(\boldsymbol{O}_j^{t-1}|\mathcal{H}(\boldsymbol{S}^t, \boldsymbol{X}_{C_\alpha}^t, \boldsymbol{O}^t)[j], \beta_{\text{orient}}^t). \tag{6}$$

S2 | ALGORITHM DETAILS FOR JOINTDIFF AND JOINTDIFF-X

S2.1 | Architecture of Graph Encoder in Reverse Diffusion

Algorithm 1 GAEncoder $(\boldsymbol{S}, \boldsymbol{X}_{C_\alpha}, \boldsymbol{O})$

Feature Collection
$\boldsymbol{T}$ = BackboneReconstruct$(\boldsymbol{S}, \boldsymbol{X}_{C_\alpha}, \boldsymbol{O})$,
$\boldsymbol{H}_{\text{node}}$ = NodeEmbed$(\boldsymbol{S}, \boldsymbol{T})$,
$\boldsymbol{H}_{\text{pair}}$ = PairEmbed$(\boldsymbol{S}, \boldsymbol{T})$,
Message Passing
$\boldsymbol{H} = \boldsymbol{H}_{\text{node}}$,
for $i = 1, \ldots, NumLayer$ **do**
    $\boldsymbol{H}$ = GABlock$_i(\boldsymbol{H}, \boldsymbol{H}_{\text{pair}}, \boldsymbol{X}_{C_\alpha}, \boldsymbol{O})$
end for
return $\boldsymbol{H}$

Node embedding and edge (pair) embedding algorithms are detailed as follows.

Algorithm 2 NodeEmbed $(\boldsymbol{S}, \boldsymbol{T})$

$\boldsymbol{H}_{\text{seq}}$ = Embed$_{\text{aa}}(\boldsymbol{S})$,
$\boldsymbol{X}_{C_\alpha}, \boldsymbol{X}_{\text{others}}$ = AtomSplit$(\boldsymbol{T})$,
$\boldsymbol{H}_{\text{coor}}$ = Global2Local(3DBasis$(\boldsymbol{X}_{\text{others}}), \boldsymbol{X}_{C_\alpha}, \boldsymbol{T})$,
$\boldsymbol{H}_{\text{dihedral}}$ = Embed$_{\text{aa}}$(GetDihedralAngles$(\boldsymbol{T})$),
$\boldsymbol{H}_{\text{node}}$ = MLP(Concat$(\boldsymbol{H}_{\text{seq}}, \boldsymbol{H}_{\text{coor}}, \boldsymbol{H}_{\text{dihedral}})$),
return $\boldsymbol{H}_{\text{node}}$

Algorithm 3 PairEmbed $(\boldsymbol{S}, \boldsymbol{T})$

$\boldsymbol{H}_{\text{aa-pair}}$ = Embed$_{\text{aa-pair}}(\boldsymbol{S})$,
$\boldsymbol{D}$ = PairWiseDistance$(\boldsymbol{T})$,
$\boldsymbol{H}_{\text{dist}}$ = Embed$_{\text{dist}}$(Kernel$_{\text{Gaussian}}(\boldsymbol{D})$),
$\boldsymbol{H}_{\text{rel-pos}}$ = Embed$_{\text{rel-pos}}$(PairWiseRelativePosition$(\boldsymbol{T})$),
$\boldsymbol{H}_{\text{dihedral}}$ = Embed$_{\text{dihedral}}$(PairWiseDihedral$(\boldsymbol{T})$),
$\boldsymbol{H}_{\text{pair}}$ = MLP(Concat$(\boldsymbol{H}_{\text{aa-pair}}, \boldsymbol{H}_{\text{rel-pos}}, \boldsymbol{H}_{\text{dist}}, \boldsymbol{H}_{\text{dihedral}})$)
return $\boldsymbol{H}_{\text{pair}}$

GABlock used in GAEncoder is detailed as follows:

Algorithm 4 GABlock ($\boldsymbol{H}_{\text{node}}, \boldsymbol{H}_{\text{pair}}, \boldsymbol{X}_{C_\alpha}, \boldsymbol{O}, \lambda = 9$)

Attention Weights

$\boldsymbol{A}_{\text{node}} = \frac{(\boldsymbol{H}_{\text{node}}\boldsymbol{W}^q)(\boldsymbol{H}_{\text{node}}\boldsymbol{W}^k)^T}{\sqrt{d}}$

$\boldsymbol{A}_{\text{pair}} = \boldsymbol{H}_{\text{pair}} \cdot \boldsymbol{W}^{\text{pair}}$,

$\boldsymbol{query}^s = \text{Local2Global}(\boldsymbol{O}, \boldsymbol{X}_{C_\alpha}, \boldsymbol{H}_{\text{node}}\boldsymbol{W}^{\text{sq}})$,

$\boldsymbol{key}^s = \text{Local2Global}(\boldsymbol{O}, \boldsymbol{X}_{C_\alpha}, \boldsymbol{H}_{\text{node}}\boldsymbol{W}^{\text{sk}})$,

$\boldsymbol{D}_{\text{sq}} = \text{Sum}((\boldsymbol{query}^s - \boldsymbol{key}^s)^2, \text{dim} = -1)$

$\boldsymbol{A}_{\text{spatial}} = -\frac{\text{SoftPlus}(\boldsymbol{W}^s) \times \sqrt{\frac{1}{4.5 \cdot \lambda}}}{2} \boldsymbol{D}_{sq}$

$\boldsymbol{A} = \text{softmax}(\boldsymbol{A}_{\text{node}} + \boldsymbol{A}_{\text{pair}} + \boldsymbol{A}_{\text{spatial}})$

Aggregation

$\boldsymbol{H}_{\text{node2node}} = \boldsymbol{A}\boldsymbol{H}_{\text{node}}\boldsymbol{W}^{\text{n2n}}$,

$\boldsymbol{H}_{\text{pair2node}} = \text{Sum}(\boldsymbol{A}\boldsymbol{H}_{\text{pair}}\boldsymbol{W}^{\text{p2n}}, \text{dim} = 2)$,

$\boldsymbol{F}^{\text{point}} = \text{Local2Global}(\boldsymbol{O}, \boldsymbol{X}_{C_\alpha}, \boldsymbol{A}(\boldsymbol{H}_{\text{node}}\boldsymbol{W}^{\text{s2n}}))$,

$\boldsymbol{F}^{\text{point}} = \text{Global2Local}(\boldsymbol{O}, \boldsymbol{X}_{C_\alpha}, \text{Sum}(\boldsymbol{F}^{\text{point}}, \text{dim} = 2))$,

$\boldsymbol{F}^{\text{point}} = \text{Norm}(\boldsymbol{F}^{\text{point}}, \text{dim} = 1)$,

$\boldsymbol{F}^{\text{dir}} = \text{Reshape}(\text{Normalize}(\boldsymbol{F}^{\text{point}}, \text{dim} = 1))$

$\boldsymbol{H}_{\text{spatial2node}} = \text{Concat}(\boldsymbol{F}^{\text{point}}, \boldsymbol{F}^{\text{dist}}, \boldsymbol{F}^{\text{dir}})$,

$\boldsymbol{H} = \text{Linear}(\text{Concat}(\boldsymbol{H}_{\text{node2node}}, \boldsymbol{H}_{\text{pair2node}}, \boldsymbol{H}_{\text{spatial2node}}))$,

$\boldsymbol{H} = \text{LayerNorm}(\boldsymbol{H}_{\text{node}} + \boldsymbol{H})$,

$\boldsymbol{H} = \text{LayerNorm}(\boldsymbol{H} + \text{MLP}(\boldsymbol{H}))$,

return $\boldsymbol{H}$

S2.2 | Training

Algorithm 5 Training Iteration of JointDiff and JointDiff-x

Require: $\boldsymbol{S}^0 = [\boldsymbol{s}_1^0, ..., \boldsymbol{s}_L^0] = \boldsymbol{S} \in \mathbb{R}^{L\times 20}$ where each $\boldsymbol{s} = \text{OneHot}(aa) \in \{0,1\}^{1\times 20}$, $aa \in \boldsymbol{w}$, and $|\boldsymbol{w}| = 20$, $\boldsymbol{X}^0 = [\boldsymbol{x}_1^0, ..., \boldsymbol{x}_L^0] = \boldsymbol{X}_{C_\alpha} \cdot sw \in \mathbb{R}^{L\times 3}$, $\boldsymbol{O}^0 = [\boldsymbol{O}_1^0, ..., \boldsymbol{O}_L^0] = \boldsymbol{O} \in (SO(3))^L$, diffusion steps T, schedulers $\bar{\alpha}_{\text{type}}^t$, $\bar{\alpha}_{\text{coor}}^t$ and $\bar{\alpha}_{\text{orient}}^t$ for $t = 1, ..., T$.

Forward diffusion:
$t \sim \text{Uniform(1, T)}, j = 1, \ldots, L$,
$\boldsymbol{s}_j^t \sim \text{Multinomial}(\bar{\alpha}_{\text{type}}^t \cdot \text{onehot}(\boldsymbol{s}_j^0) + (1 - \bar{\alpha}_{\text{type}}^t) \cdot \frac{1}{20} \cdot \mathbf{1})$,
$\boldsymbol{x}_j^t = \sqrt{\bar{\alpha}_{\text{coor}}^t} \cdot \boldsymbol{x}_j^0 + (1 - \bar{\alpha}_{\text{coor}}^t)\epsilon_j$, $\boldsymbol{\epsilon} \sim \mathcal{N}(\mathbf{0}, \boldsymbol{I})$,
$\boldsymbol{O}_j^t \sim \mathcal{IG}_{SO(3)}(\boldsymbol{O}_j^t | \text{ScaleRot}(\bar{\alpha}_{\text{orient}}^t, \boldsymbol{O}_j^0), 1 - \bar{\alpha}_{\text{orient}}^t)$.
Reverse diffusion:
$\boldsymbol{H}^t \in \mathbb{R}^{L\times d} = \text{GAEncoder}(\boldsymbol{S}^t, \boldsymbol{X}^t, \boldsymbol{O}^t)$,
$\boldsymbol{h}_j^t = \text{Concat}(\boldsymbol{h}_j^t, \beta^t, \sin(\beta^t), \cos(\beta^t)), j = 1, \ldots, L$,
$\hat{\boldsymbol{O}}^{t-1} = \text{Projector}_{\text{orient}}(\boldsymbol{H}^t, \boldsymbol{O}^t) \in SO(3)^L$,
Objective function:
if JointDiff **then**
  $p(\boldsymbol{S}^{t-1} | \boldsymbol{S}^t, \boldsymbol{X}^t, \boldsymbol{O}^t) = \text{Projector}_{\text{type}}(\boldsymbol{H}^t) \in (0,1)^{L\times 20}$,
  $\hat{\epsilon} = \text{Projector}_{\text{coor}}(\boldsymbol{H}^t, \boldsymbol{O}^t) \in \mathbb{R}^{L\times 3}$,
  $\mathcal{L}_{\text{type}}^t = \frac{1}{L}\sum_{j=1}^L D_{KL}(q(\boldsymbol{s}_j^{t-1} | \boldsymbol{s}_j^t, \boldsymbol{s}_j^0) || p(\boldsymbol{s}_j^{t-1} | \boldsymbol{S}^t, \boldsymbol{X}^t, \boldsymbol{O}^t))$,
  $\mathcal{L}_{\text{coor}}^t = \frac{1}{L}\sum_{j=1}^L \|\epsilon_j - \hat{\epsilon}_j\|^2$,
else if JointDiff-x **then**
  $p(\boldsymbol{S}^0 | \boldsymbol{S}^t, \boldsymbol{X}^t, \boldsymbol{O}^t) = \text{Projector}_{\text{type}}(\boldsymbol{H}^t) \in (0,1)^{L\times 20}$,
  $\widehat{\boldsymbol{X}}^0 = \text{Projector}_{\text{coor}}(\boldsymbol{H}^t, \boldsymbol{O}^t) \in \mathbb{R}^{L\times 3}$,
  $\mathcal{L}_{\text{type}}^t = \frac{1}{L}\sum_{j=1}^L \text{CrossEntropy}(\boldsymbol{s}_j^0, p(\boldsymbol{s}_j^0 | \boldsymbol{S}^t, \boldsymbol{X}^t, \boldsymbol{O}^t))$,
  $\mathcal{L}_{\text{coor}}^t = \frac{1}{L}\sum_{j=1}^L \|\boldsymbol{x}_j^0 - \hat{\boldsymbol{x}}_j^0\|^2$,
end if
$\mathcal{L}_{\text{orient}}^t = \frac{1}{L}\sum_{j=1}^L \|(\boldsymbol{O}_j^0)^T \hat{\boldsymbol{O}}_j^{t-1} - \boldsymbol{I}\|_F^2$,
$\mathcal{L}_{\text{multi}} = \mathbb{E}_{t\sim\text{Uniform}(1,...,T)}[\mathcal{L}_{\text{type}}^t + \mathcal{L}_{\text{coor}}^t + \mathcal{L}_{\text{orient}}^t]$.

Coordinate Losses.

For **JointDiff-x**, the default coordinate loss $\mathcal{L}_{\text{coor}}$ is the mean squared error (MSE), as defined above. Another widely used SE(3)-invariant loss is the *Frame Aligned Point Error (FAPE)*, originally introduced in AlphaFold2 [5], which demonstrated strong performance in monomer structure prediction.

AlphaFold2 models each residue using idealized backbone geometry (i.e., fixed bond lengths and angles), and predicts a local frame $\boldsymbol{T}_j = (\boldsymbol{R}_j, \boldsymbol{t}_j)$ for each residue $j = 1, \ldots, L$, where $\boldsymbol{R}_j \in \mathbb{R}^{3\times 3}$ is a rotation matrix and $\boldsymbol{t}_j \in \mathbb{R}^3$ is a translation vector. These frames are used to reconstruct the full-atom 3D structure, which is further refined using predicted torsion angles for side chains.

In our case of *backbone-only design*, we focus solely on the backbone frames. Here, $\boldsymbol{R}_j$ corresponds to the local orientation $\boldsymbol{O}_j$, and $\boldsymbol{t}_j$ can be represented by the Cα coordinates $\boldsymbol{x}_j$. Based on this formulation, we also experimented with using FAPE as the coordinate loss, as described in Algorithm 6. We use hats over symbols and superscripts 0 to indicate the model estimations and the ground truth values, respectively.

Algorithm 6 Compute the Frame Aligned Point Error (FAPE)

def FAPE $\left(\{\widehat{\boldsymbol{T}}_j\}, \{\hat{\boldsymbol{x}}_j\}, \{T_j^0\}, \{\boldsymbol{x}_j^0\}, Z = 10\text{Å}, d_{\text{clamp}} = 10\ \text{Å}, \epsilon = 10^{-4}\ \text{Å}^2\right)$:

1: $\hat{\boldsymbol{x}}_{ij} = \widehat{\boldsymbol{T}}_i^{-1} \circ \hat{\boldsymbol{x}}_j = \widehat{\boldsymbol{O}}_i^{-1} \cdot (\hat{\boldsymbol{x}}_j - \hat{\boldsymbol{x}}_i)$

2: $\boldsymbol{x}_{ij}^0 = (\boldsymbol{T}_i^0)^{-1} \circ \boldsymbol{x}_j^0 = (\boldsymbol{O}_i^0)^{-1} \cdot (\boldsymbol{x}_j^0 - \boldsymbol{x}_i^0)$

3: $d_{ij} = \sqrt{\left\|\hat{\boldsymbol{x}}_{ij} - \boldsymbol{x}_{ij}^0\right\|^2 + \epsilon}$

4: $\mathcal{L}_{\text{FAPE}} = \frac{1}{Z}\text{mean}_{\{(i,j)\}}\left(\min(d_{ij}, d_{\text{clamp}})\right)$

5: **Return** $\mathcal{L}_{\text{FAPE}}$

S2.3 | Inference

Algorithm 7 Inference Steps of JointDiff and JointDiff-x

Initialization:
$\hat{s}_j^T \sim \text{Uniform}(20)$, $\hat{\boldsymbol{x}}_j^T \sim \mathcal{N}(\mathbf{0}, \boldsymbol{I})$, $\widehat{\boldsymbol{O}}_j^T \sim \mathcal{IG}_{SO(3)}(\mathbf{0}, \boldsymbol{I})$, $j = 1, \ldots, L$.
for t = T, T-1, ..., 1 **do**
    $\boldsymbol{H}^t \in R^{L \times d} = \text{GAEncoder}(\widehat{\boldsymbol{S}}^t, \widehat{\boldsymbol{X}}^t, \widehat{\boldsymbol{O}}^t)$,
    $\boldsymbol{h}_j^t = \text{Concat}(\boldsymbol{h}_j^t, \beta^t, \sin(\beta^t), \cos(\beta^t)), j = 1, \ldots, L$,
    if JointDiff **then**
        $\widehat{\boldsymbol{S}}^{t-1} \sim p(\boldsymbol{S}^{t-1} | \boldsymbol{S}^t = \widehat{\boldsymbol{S}}^t, \boldsymbol{X}^t = \widehat{\boldsymbol{X}}^t, \boldsymbol{O}^t = \widehat{\boldsymbol{O}}^t) = \text{Projector}_{\text{type}}(\boldsymbol{H}^t) \in (0,1)^{L \times 20}$,
        $\hat{\epsilon} = \text{Projector}_{\text{coor}}(\boldsymbol{H}^t, \widehat{\boldsymbol{O}}^t) \in (0,1)^{L \times 3}$,
        $\boldsymbol{\mu}_j = \frac{1}{\sqrt{\alpha_{\text{coor}}^t}}(\hat{\boldsymbol{x}}_j^t - \frac{\beta_{\text{coor}}^t}{\sqrt{1-\alpha_{\text{coor}}^t}}\hat{\boldsymbol{\epsilon}}_j)$,
        $\hat{\boldsymbol{x}}_j^{t-1} \sim \mathcal{N}(\boldsymbol{x} | \boldsymbol{\mu}_j, \beta_{\text{coor}}^t \boldsymbol{I})$,
        $\widehat{\boldsymbol{O}}^{t-1} = \text{Projector}_{\text{orient}}(\boldsymbol{H}^t, \widehat{\boldsymbol{O}}^t) \in (SO(3))^L$,
    else if JointDiff-x **then**
        $\widehat{\boldsymbol{S}}^0 \sim p(\boldsymbol{S}^0 | \boldsymbol{S}^t = \widehat{\boldsymbol{S}}^t, \boldsymbol{X}^t = \widehat{\boldsymbol{X}}^t, \boldsymbol{O}^t = \widehat{\boldsymbol{O}}^t) = \text{Projector}_{\text{type}}(\boldsymbol{H}^t) \in (0,1)^{L \times 20}$,
        $\widehat{\boldsymbol{X}}^0 = \text{Projector}_{\text{coor}}(\boldsymbol{H}^t, \widehat{\boldsymbol{O}}^t) \in (0,1)^{L \times 3}$
        $\widehat{\boldsymbol{O}}^0 = \text{Projector}_{\text{orient}}(\boldsymbol{H}^t, \widehat{\boldsymbol{O}}^t) \in (SO(3))^L$,
        if $t \geq 2$ **then**
            $\hat{s}_j^{t-1} \sim \text{Multinomial}\left(\bar{\alpha}_{\text{type}}^{t-1} \cdot \text{onehot}(\widehat{aa}_j^0) + (1 - \bar{\alpha}_{\text{type}}^{t-1}) \cdot \frac{1}{20} \cdot \mathbf{1}\right)$,
            $\hat{\boldsymbol{x}}_j^{t-1} = \sqrt{\bar{\alpha}_{\text{coor}}^{t-1}} \cdot \hat{\boldsymbol{x}}_j^0 + (1 - \bar{\alpha}_{\text{coor}}^{t-1})\epsilon_j, \quad \boldsymbol{\epsilon} \sim \mathcal{N}(\mathbf{0}, \boldsymbol{I})$,
            $\widehat{\boldsymbol{O}}_j^{t-1} \sim \mathcal{IG}_{SO(3)}\left(\boldsymbol{O}_j^{t-1} | \text{ScaleRot}(\bar{\alpha}_{\text{orient}}^{t-1}, \widehat{\boldsymbol{O}}_j^0), 1 - \bar{\alpha}_{\text{orient}}^{t-1}\right)$.
        end if
    end if
    Unnormarlization: $\widehat{\boldsymbol{X}}^0 = \widehat{\boldsymbol{X}}^0 \cdot \frac{1}{sw}$,
end for
return $\widehat{\boldsymbol{S}}^0, \widehat{\boldsymbol{X}}^0, \widehat{\boldsymbol{O}}^0$

S2.4 | Guided Inference

Algorithm 8 Inference Steps of JointDiff-x with CATH guidance

Initialization:
$\hat{s}_j^T \sim \text{Uniform}(20)$, $\hat{\boldsymbol{x}}_j^T \sim \mathcal{N}(\mathbf{0}, \boldsymbol{I})$, $\widehat{\boldsymbol{O}}_j^T \sim \mathcal{IG}_{SO(3)}(\mathbf{0}, \boldsymbol{I}), j = 1, \ldots, L.$
for t = T, T-1, ..., 1 **do**
  $\boldsymbol{H}^t = \text{GAEncoder}(\widehat{\boldsymbol{S}}^t, \widehat{\boldsymbol{X}}^t, \widehat{\boldsymbol{O}}^t)$,
  $\boldsymbol{h}_j^t = \text{Concat}(\boldsymbol{h}_j^t, \beta^t, \sin(\beta^t), \cos(\beta^t)), j = 1, \ldots, L$
  $\widehat{\boldsymbol{S}}^0 \sim p(\boldsymbol{S}^0 | \boldsymbol{S}^t = \widehat{\boldsymbol{S}}^t, \boldsymbol{X}^t = \widehat{\boldsymbol{X}}^t, \boldsymbol{O}^t = \widehat{\boldsymbol{O}}^t) = \text{Projector}_{\text{type}}(\boldsymbol{H}^t) \in (0, 1)^{L \times 20}$,
  $\widehat{\boldsymbol{X}}^0 = \text{Projector}_{\text{coor}}(\boldsymbol{H}^t, \widehat{\boldsymbol{O}}^t) \in (0, 1)^{L \times 3}$
  $\widehat{\boldsymbol{O}}^0 = \text{Projector}_{\text{orient}}(\boldsymbol{H}^t, \widehat{\boldsymbol{O}}^t) \in (SO(3))^L$,
  $w^t = \lambda\left(\frac{t}{T}\right)^2; \ \mathcal{L}_{\text{cls}}(\cdot) = -\log f_\phi\left(y^{\text{target}} \,\middle|\, \boldsymbol{X}, \boldsymbol{O}\right)$
  $\boldsymbol{g}^t(\cdot) = \nabla_{\boldsymbol{X}} \mathcal{L}_{\text{cls}}(\cdot); \ \widehat{\boldsymbol{X}}^0 \leftarrow \widehat{\boldsymbol{X}}^0 - \eta^t w^t \boldsymbol{g}^t(\boldsymbol{X} = \widehat{\boldsymbol{X}}^0, \boldsymbol{O} = \widehat{\boldsymbol{O}}^0)$
  if $t \geq 2$ **then**
    $\hat{s}_j^{t-1} \sim \text{Multinomial}\left(\bar{\alpha}_{\text{type}}^{t-1} \cdot \text{onehot}(\widehat{aa}_j^0) + (1 - \bar{\alpha}_{\text{type}}^{t-1}) \cdot \frac{1}{20} \cdot \mathbf{1}\right)$,
    $\hat{\boldsymbol{x}}_j^{t-1} = \sqrt{\bar{\alpha}_{\text{coor}}^{t-1}} \cdot \hat{\boldsymbol{x}}_j^0 + (1 - \bar{\alpha}_{\text{coor}}^{t-1})\epsilon_j, \quad \epsilon \sim \mathcal{N}(\mathbf{0}, \boldsymbol{I})$,
    $\widehat{\boldsymbol{O}}_j^{t-1} \sim \mathcal{IG}_{SO(3)}\left(\boldsymbol{O}_j^{t-1} | \text{ScaleRot}(\bar{\alpha}_{\text{orient}}^{t-1}, \widehat{\boldsymbol{O}}_j^0), 1 - \bar{\alpha}_{\text{orient}}^{t-1}\right)$.
  end if
  Unnormarlization: $\widehat{\boldsymbol{X}}^0 = \widehat{\boldsymbol{X}}^0 \cdot \frac{1}{sw}$,
end for
return $\widehat{\boldsymbol{S}}^0$, $\widehat{\boldsymbol{X}}^0$, $\widehat{\boldsymbol{O}}^0$

S3 | ADDITIONAL RESULTS

S3.1 | Performance of the ConfidenceNet

We evaluated the performance of our confidence net. While the models were trained with binary classification and regression tasks separately, and our goal is to select qualified samples, we evaluated the confidence models by checking how the selected samples would align with qualified one, i.e. we did binary classification tasks on our test set. For the regression models, we use the same criteria to determine the qualified samples as we assigned the labels. We applied accuracy, F1-score and Spearman correllation as the evaluation metrics. From Table S1, we find that the balanced training was generally helpful for the binary models, and normalization can also improve the performance on regression models.

Model	foldability			designability			str2seq			seq2str		
	acc. ↑	F1 ↑	spr. ↑	acc. ↑	F1 ↑	spr. ↑	acc. ↑	F1 ↑	spr. ↑	acc. ↑	F1 ↑	spr. ↑
Binary	0.956	0.943	0.755	0.888	0.891	0.913	0.970	0.958	0.511	0.916	0.903	0.880
Binary-balance	0.952	0.942	0.776	0.891	0.895	0.915	0.976	0.963	0.519	0.917	0.904	0.886
Regression	0.952	0.941	0.818	0.890	0.888	0.932	0.978	0.958	0.801	0.914	0.899	0.895
Regression-norm	0.955	0.946	0.850	0.900	0.905	0.934	0.979	0.962	0.844	0.915	0.904	0.906

TABLE S1 Performance of the confidence net on our test set.

S3.2 | Structure Data Distribution

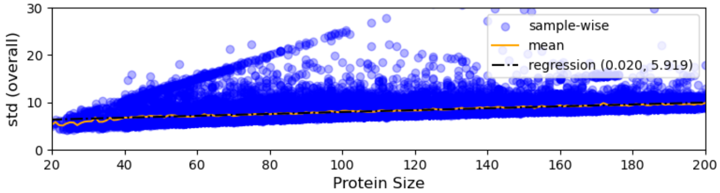

FIGURE S1 Distribution of sample-wise standard deviations (std) along protein size in the CATH dataset. The std value increased almost in a linear manner as the protein length grows.

S3.3 | Detailed Evaluation on the Impact of Structure Normalization

To understand the role of structure normalization in JointDiff training, we tested both centering and scaling strategies. Scaling was applied in two forms: length-independent ($sw \in [1, 0.01]$) and

length-dependent ($sw = \frac{1}{c(aL+b)}$, with $c \in [1, 50]$). Centering improved designability for certain sw values by removing location bias. Scaling had a stronger effect: self-consistency of JointDiff-designed structures increased from 0.300 ($sw = 1$) to 0.692 ($sw = 0.02$). Sequence-to-structure cross-consistency also rose significantly, while structure-to-sequence consistency slightly declined. Length-dependent scaling did not yield further improvements.

For JointDiff-x, normalization had less influence due to its direct structure prediction via ReverseNet. Nonetheless, $sw = 0.02$ was adopted for consistency and performance. Full results are summarized in Table S2.

JointDiff Configuration		Self-Consistency ↑ seq (fold.)	Self-Consistency ↑ str (design.) (TM-score)	Cross-Consistency ↑ str2seq	Cross-Consistency ↑ seq2str (TM-score)	Clash ↓ str
uncentered structures	sw=1.0	0.199	0.300	0.257	0.267	**8.507**
	sw=0.1	0.228	0.390	0.194	0.354	11.954
	sw=0.05	0.223	0.509	0.197	0.467	16.308
	sw=0.02	0.221	0.629	0.198	0.574	45.813
	sw=0.01	0.227	0.570	0.199	0.529	96.039
**centered structures**	sw=1.0	0.198	0.300	**0.259**	0.274	13.112
	sw=0.1	0.232	0.548	0.206	0.428	14.131
	sw=0.05	0.214	0.500	0.195	0.457	20.112
	sw=**0.02**	0.217	**0.692**	0.199	**0.591**	30.26
	sw=0.01	0.218	0.581	0.194	0.549	80.29
centered structures	length-dep. sw (c=1)	0.229	0.301	0.191	0.277	8.790
	length-dep. sw (c=10)	0.224	0.494	0.182	0.524	65.623
	length-dep. sw (c=20)	0.220	0.491	0.167	0.525	124.516
	length-dep. sw (c=50)	**0.233**	0.324	0.157	0.440	224.457

TABLE S2 Impact of centering and scaling structure data on JointDiff-designed sequences and structures, specifically, their self-consistency (foldability for sequence and designability for structure) and cross-consistency (sequence-to-structure and structure-to-sequence consistency). The noise-prediction (ϵ-prediction) loss was used. Boldfaced configurations (centerted structures and $sw = 0.02$) are chosen for the final JointDiff model. Boldfaced entries highlight the top results for each evaluation metric.

S3.4 | Distributions of Consistency Scores

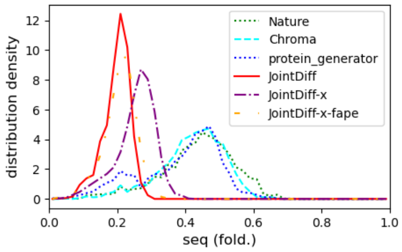

(a) Sequence Foldability

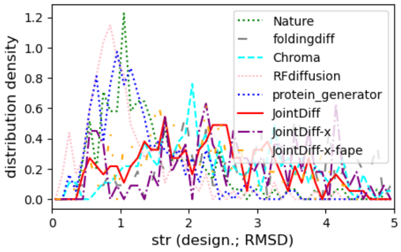

(b) Structure Designability

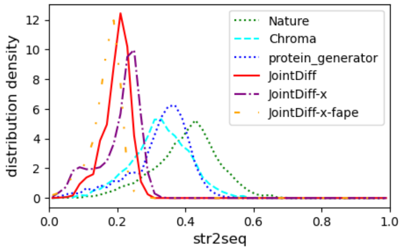

(c) Structure-to-Sequence Cross-consistency

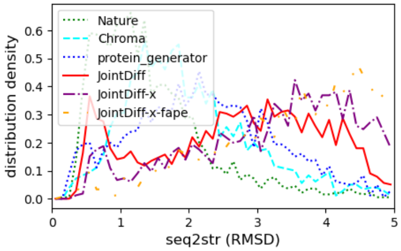

(d) Sequence-to-Structure Cross-consistency

FIGURE S2 Evaluating natural and various model-designed proteins in distributions over four computational assessment scores.

S3.5 | Novelty, Diversity, and Sanity Checks

Model	Sequence			Structure		
	Diversity ↑	Novelty ↑	5-repeats ↓	Diversity ↑	Novelty ↑	Clash ↓
Nature	94.8%	-	0.07%	84.4%	-	0.047
FoldingDiff	-	-	-	100%	100%	7.86
RFdiffusion	-	-	-	99.2%	99.8%	1.22
Chroma+Potts	100%	100%	0.59%	100%	97.0%	5.28
ProteinGenerator	99.8%	100%	0.52%	97.8%	98.0%	19.22
JointDiff	100%	100%	0.27%	99.6%	100%	30.26
JointDiff-x	100%	100%	0.38%	96.0%	99.8%	8.13
JointDiff-x (FAPE)	100%	100%	0.08%	99.8%	99.6%	10.50

TABLE S3 Diversity and novelty (at default similarity thresholds) as well as sanity checks for designed sequences or structures.

S3.6 | Computational Evaluation of JointDiff-x Models on A Curated Test Set for GFP

Model		Self-Consistency ↑		Cross-Consistency ↑	
		seq (fold.)	str (design.) (TM-score)	str2seq	seq2str (TM-score)
RFdiffusion		-	0.869	-	-
JointDiff-x (FAPE) (General)	CATH4.4	0.264	0.428	0.234	0.332
	AFDB-L300	0.236	0.450	0.207	0.316
	AFDB-L300 (sepa.)	0.247	0.458	0.246	0.304
JointDiff-x (FAPE) (Fine-Tuned)		0.245	0.563	0.187	0.263

TABLE S4 Self-consistencies and cross-consistencies on the motif-scaffolding task of GFP.

S3.7 | Supplement Results for Experimental Validation of GFPs

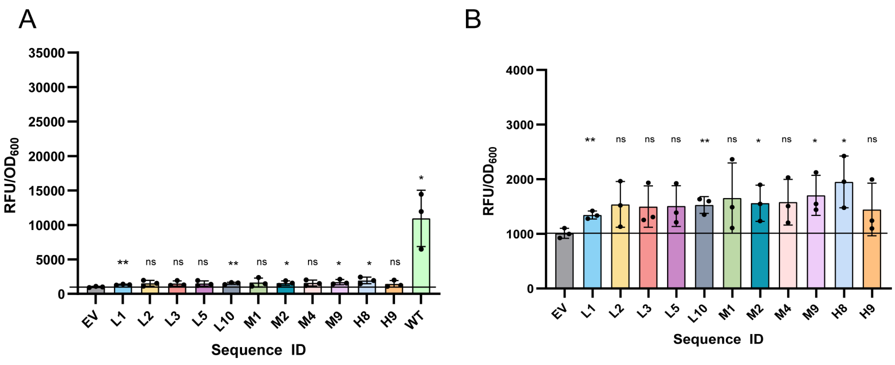

FIGURE S3 (A) Fluorescence intensity of *E. coli* whole-cells expressing the designed GFP variants 24 h after inoculation. *E. coli* cells expressing avGFP (WT) served as the positive control, and cells harboring a plasmid without the GFP gene (empty vector, EV) was used as a negative control. (B) A zoomed-in view of the data presented in 1(A), showing the relative fluorescence of each design variant when compared to the empty vector (EV). Experiments were conducted in triplicate, and data shown are mean values (± standard deviation). Statistical significance was evaluated using unpaired Student's t-tests. $^{*}p < 0.05$; $^{**}p < 0.01$, ns – Not significant.

references

[1] Hoogeboom E, Nielsen D, Jaini P, Forré P, Welling M. Argmax flows and multinomial diffusion: Learning categorical distributions. Advances in Neural Information Processing Systems 2021;34:12454–12465.

[2] Ho J, Jain A, Abbeel P. Denoising diffusion probabilistic models. Advances in Neural Information Processing Systems 2020;33:6840–6851.

[3] Leach A, Schmon SM, Degiacomi MT, Willcocks CG. Denoising diffusion probabilistic models on so (3) for rotational alignment. In: ICLR 2022 Workshop on Geometrical and Topological Representation Learning; 2022. .

[4] Gallier J, Xu D. Computing exponentials of skew-symmetric matrices and logarithms of orthogonal matrices. International Journal of Robotics and Automation 2003;18(1):10–20.

[5] Jumper J, Evans R, Pritzel A, Green T, Figurnov M, Ronneberger O, et al. Highly accurate protein structure prediction with AlphaFold. Nature 2021;596(7873):583–589.
